# Sustainable production of hydrogen with high purity from methanol and water at low temperatures

**DOI:** 10.1038/s41467-022-33186-z

**Published:** 2022-09-21

**Authors:** Sai Zhang, Yuxuan Liu, Mingkai Zhang, Yuanyuan Ma, Jun Hu, Yongquan Qu

**Affiliations:** 1 School of Chemistry and Chemical Engineering, Northwestern Polytechnical University, 710072 Xian, China; 2 Research & Development Institute of Northwestern Polytechnical University in Shenzhen, 518057 Shenzhen, China; 3 Center for Applied Chemical Research, Frontier Institute of Science and Technology, Xian Jiaotong University, 710049 Xian, China; 4 School of Chemical Engineering, Northwest University, 710069 Xian, China

**Keywords:** Heterogeneous catalysis, Hydrogen energy, Chemical engineering

## Abstract

Carbon neutrality initiative has stimulated the development of the sustainable methodologies for hydrogen generation and safe storage. Aqueous-phase reforming methanol and H_2_O (APRM) has attracted the particular interests for their high gravimetric density and easy availability. Thus, to efficiently release hydrogen and significantly suppress CO generation at low temperatures without any additives is the sustainable pursuit of APRM. Herein, we demonstrate that the dual-active sites of Pt single-atoms and frustrated Lewis pairs (FLPs) on porous nanorods of CeO_2_ enable the efficient additive-free H_2_ generation with a low CO (0.027%) through APRM at 120 °C. Mechanism investigations illustrate that the Pt single-atoms and Lewis acidic sites cooperatively promote the activation of methanol. With the help of a spontaneous water dissociation on FLPs, Pt single-atoms exhibit a significantly improved reforming of *CO to promote H_2_ production and suppress CO generation. This finding provides a promising path towards the flexible hydrogen utilizations.

## Abstract (web summary)

Carbon neutrality initiative has stimulated the development of the sustainable methodologies for hydrogen generation and safe storage. Here the authors demonstrate that the dual-active sites of Pt single-atoms and frustrated Lewis pairs on porous nanorods of CeO2 enable the efficient additive-free H2 generation with a low CO generation through reforming of methanol and water.

## Introduction

Developing hydrogen (H_2_) generation and storage technologies to sustainably supply such a clean power energy resource with high calorific value is crucial to alleviating the global energy/environmental crisis and realizing the carbon neutrality initiative^1,2^. However, the inflammability and explosibility of H_2_, as well as its extremely high liquefied pressure (700 psi), technically and economically raise the grand challenges in cost, safety, and reliability during the operations for hydrogen transportation and storage. Afterward, various liquid organic carriers have been proposed to store hydrogen in the dense liquid phase, which offers a promising methodology with high capacity and safe re-distributions on demands. Among various liquid hydrogen carriers (formic acid^3–5^, *N*-heteroarenes^6–8^, cyclohexane^9^, etc.), methanol, as a sustainable, inexpensive, and readily available hydrogen source derived from biomass and/or CO_2_ hydrogenation, can give 18.8 wt.% H_2_ gravimetric density via reforming with H_2_O to release three equivalent amounts of H_2_. Combining its mature and safe technology for storage and transportation, methanol has been recognized as one of the most promising candidates among all known liquid hydrogen carriers^10,11^.

Although a homogeneous metal catalytic system with meticulously designed ligands can enable hydrogen release at temperatures below 100 °C, the large amounts of strong bases (e.g., 8 M KOH, NaOH) are generally adopted to trigger the entire catalytic process by activating CH_3_OH/H_2_O at low temperatures and maintain the catalytic activity by neutralizing the generated formic acid as well as CO_2_^10,12–14^. From the environmentally sustainable and economical perspectives, various heterogeneous catalysts, including Pt-, Ru-, Pd-based catalysts, have been developed as the additive-free catalytic system for hydrogen generation. However, the reforming of methanol and water by those catalysts generally faces two big obstacles: (1) the high temperatures (å 250 °C) to boost catalytic reaction, and (2) the low purity of H_2_ accompanied by the generation of CO at a high level^15–19^. Recent advances in developing new heterogeneous catalysts have greatly decreased the operation temperatures as low as 150 °C for the aqueous-phase reforming of methanol by using the atomically dispersed Pt on α-MoC^11^. Afterward, the further decrease of the reaction temperatures with a satisfactory H_2_ generation rate is extremely difficult and rarely realized on heterogeneous catalysts yet up to now. Therefore, developing high-efficient catalysts capable of in situ releasing of H_2_ at even lower temperatures and the suppressed CO generation is highly desirable for the large-scale production of hydrogen, bringing us a step closer to a methanol economy.

Herein, we demonstrate that the dual-active site catalysts composed of the Pt single-atoms and frustrated Lewis pairs (FLPs) on the atomically dispersed Pt anchored on porous nanorods of CeO_2_ (Pt_1_/PN-CeO_2_) enable a stabilized H_2_ generation at a low temperature of 120 °C through a base-free aqueous-phase reforming of methanol (APRM). The catalytic activity and selectivity were examined in a closed system under an initial pressure of 0.4 MPa N_2_. The turnover frequency (TOF) of Pt_1_/PN-CeO_2_ based on Pt atoms was 33 h^−1^ at 120 °C, which was comparable to or even superior to the majority of homogeneous and heterogeneous catalysts operated in the presence/absence of additives/bases under similar reaction temperatures. Encouragingly, the selectivity of CO by-product, a harmful molecule for downstream metal catalysts, was significantly suppressed below 0.03% under such mild reaction conditions.

## Results

### Catalyst design

**Fig. 1:**
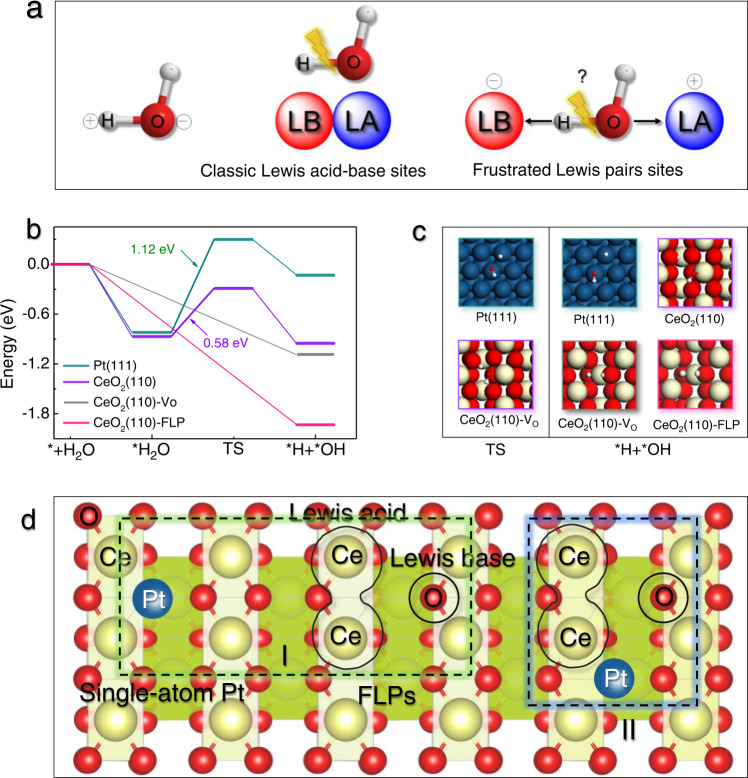
Catalyst design. **a** Scheme of H_2_O activation on classic Lewis acid-base sites and FLPs. **b** Energy barrier and **c** adsorption configuration of H_2_O activation on various actives sites. **d** The designed dual-active sites of single-atom Pt and FLPs. Note: The dark bule, red, and yellow balls respective the Pt, O, and Ce atoms, respectively. The abbreviation of FLP is the frustrated Lewis pairs.

To achieve high activity of catalysts for reforming methanol and water at low temperatures, the key steps are analyzed initially. Theoretically, H_2_ generation from methanol and water generally involves three reaction steps: (1) CH_3_OH dissociation into *CO and *H intermediates; (2) H_2_O activation into *OH and *H intermediates; and (3) transformation of *CO and *OH into CO_2_(g) and *H through a water-gas shift reaction^20^. Therefore, the ideal scenario requires a catalyst with the effective activation of both CH_3_OH and H_2_O to guarantee efficient H_2_ generation at low temperatures. However, there is difficult to be achieved on a single type of active site^11,19^. The principle behind this phenomenon is that the metal active sites with high capability for CH_3_OH activation generally exhibit a low ability for H_2_O dissociation^20,21^. Different from CH_3_OH activation on metal surfaces, the H_2_O activation can be significantly promoted on reducible metal oxides via O atom interaction with Lewis acidic center (metal site), and then one of H atoms transfers to the adjacent Lewis basic center (lattice oxygen site, Fig. 1a)^20,22–26^. Therefore, the construction of the dual-active sites for the respective methanol and water activation potentially provides a feasible approach to efficiently producing H_2_ at low temperatures.

Of the candidate catalysts for CH_3_OH activation, Pt is selected due to its strong capacity for the CH_3_OH dissociation into *CO and *H active intermediates^11,27^. Then, density function theory (DFT) calculations were performed to investigate the H_2_O activation on Pt (111) surface. As expected, the Pt(111) surface exhibited the kinetically unfavorable process for H_2_O activation with a derived large kinetic energy barrier of 1.12 eV (Fig. 1b, c)^11^. Therefore, similar to other metals^22^, the H_2_O dissociation is difficult to occur on Pt surface, thereby limiting the subsequent reforming of the *CO intermediates with *OH at low temperatures.

Inspired by the typical Lewis acid-base interaction for H_2_O activation (Fig. 1a), frustrated Lewis Pairs (FLPs), which are composed of a sterically hindered Lewis acid and Lewis base^28–31^, provides a promising opportunity for efficiently activating H_2_O molecule at low temperatures. In this configuration, the negative O and H atoms of H_2_O interact closely with Lewis acidic site and basic site of FLPs at the same time (Fig. 1a), respectively, revealing an attractive pathway for H_2_O activation. Recently, the all-solid FLP sites constituted by the two adjacent surface Ce^3+^ (Lewis acid sites) and the neighboring surface lattice oxygen (Lewis base sites) have been successfully constructed on the PN-CeO_2_, as shown in Supplementary Fig. 1. Due to the unique configuration of FLPs and the different formation energies of oxygen vacancy on various surfaces, CeO_2_(110) surface instead of CeO_2_(100) and CeO_2_(111) surfaces has been previously verified to exhibit the highest probability for the FLP’s construction (Supplementary Fig. 2)^32,33^. Those investigations have demonstrated that the abundant surface defects of oxygen vacancy on CeO_2_(110) benefit the formation of interfacial FLP sites. To explore their ability for H_2_O activation, DFT calculations were thereafter performed to investigate the H_2_O decomposition on CeO_2_(110) surfaces with various densities of a structural defect, i.e., ideal CeO_2_(110), CeO_2_(110) with one oxygen vacancy (CeO_2_(110)-V_O_) and CeO_2_(110) surface with FLPs sites (CeO_2_(110)-FLP).

On the ideal CeO_2_(110) surface, the H_2_O activation can easily occur with a low energy barrier of 0.58 eV (Fig. 1d, e). More importantly, the dissociation of H_2_O can be further promoted in the presence of oxygen vacancy on CeO_2_(110) surface. As shown in Fig. 1b, c, both CeO_2_(110)-V_O_ with one oxygen vacancy and CeO_2_(110)-FLP with two adjacent oxygen vacancies exhibit the adsorptive dissociation of H_2_O without a transition state in comparison with ideal CeO_2_(110). Especially, the dissociation of H_2_O on CeO_2_(110)-FLP is also the thermodynamically favored process compared with CeO_2_(110)-O_V_ active sites. Therefore, both kinetic and thermodynamic analysis reveals the FLP sites with the optimal ability to dissociate H_2_O.

Considering that the single-atom Pt active sites exhibit higher capability for CH_3_OH activation than Pt nanoparticles^11,27^, the construction of the dual-active sites of single-atom Pt and FLPs (Pt_1_-FLP) can effectively activate both H_2_O and CH_3_OH, respectively, potentially reducing the reaction temperatures of APRM. Then, DFT calculations were further used to explore the possible spatial structures of Pt atom on CeO_2_(110) surface, which delivered two configurations. As shown in Supplementary Fig. 3, the Pt atom prefers to occupy the oxygen defect of CeO_2_(110) surface owing to the lowest formation energy (1.35 eV). In this configuration, the FLP site is not affected by the single-atom Pt in the distance. Therefore, the Type **I** of Pt_1_-FLP due-active site is successfully constructed, in which Pt single-atom locates at the oxygen vacancy of CeO_2_(110) surface (Fig. 1d). In addition, the Pt single-atom can occupy one of the oxygen vacancies adjacent to the FLP site with slightly high formation energy (1.70 eV). For this configuration, the type **II** of Pt_1_-FLP dual-active site is spatially adjacent to each other, as shown in Fig. 1d and Supplementary Fig. 3. Also, the spatial and electronic configuration of FLP sites are preserved in the presence of the nearby single Pt atom.

Due to the high ability for H_2_O activation on FLP sites, the reforming of *CO with *OH on the Pt_1_-FLP dual-active site is also significantly improved through a water-gas shift reaction process. As shown in Supplementary Fig. 4, the energy barrier is only 0.89 eV on the adjacent Pt_1_-FLP dual-active site (type **II**), which is dramatically lower than that of 2.15 eV on the surface of Pt(111) surface. For the type **I**, due to the long spatial distance between Pt single-atoms and FLPs sites, the generated *OH on FLP sites could diffuse near the Pt single-atom owing to the easily occurred migration of H and O atoms on CeO_2_(110)^34–36^. Theoretically, the dual-active sites of single-atom Pt and FLPs (Fig. 1d) can significantly reduce the reaction temperature for APRM, resulting in successful H_2_ generation at low temperatures.

### Synthesis and characterizations of Pt_1_-FLP dual-active site catalysts

The open question herein is how to synthesize the Pt_1_-FLP dual-active sites to devise an efficient catalyst for H_2_ generation from APRM. A two-step hydrothermal process was used to prepare highly defective PN-CeO_2_ with a length of ~65 nm and a daimeter of ~7 nm, as revealed from the dark-field transmission electron microscopy (TEM) image (Supplementary Fig. 5a). The 0.275 nm of lattice frings spacing was consistent with the (220) crystal face of CeO_2_ (Supplementary Fig. 5b), further revealing PN-CeO_2_ along with [110] direction^37^. The specific surface area of PN-CeO_2_ was 109 m^2^ g^−1^, as derived from the N_2_ adsorption/desorption isotherm plot (Supplementary Fig. 6a). The porous structure with a size of 1.5–3.0 nm was revealed from TEM images (Supplementary Fig. 5b) as well as the Brunauer-Emmett-Teller (BET) measurements (Supplementary Fig. 6b, c). More importantly, the abundance of surface defect on PN-CeO_2_ was indexed by the 30.8% surface Ce^3+^ fraction as well as the 47.1% surface Ce^3+^-O fraction, derived from its X-ray photoelectron spectroscopy (XPS) spectrum of Ce *3d* and O *1s*, respectively (Supplementary Fig. 7 and Supplementary Table 1). Therefore, the FLP sites could be formed on the PN-CeO_2_ supports owing to the high concentration of oxygen defect on the CeO_2_(110) surface, as described in our previous reports^32,33^.

**Fig. 2:**
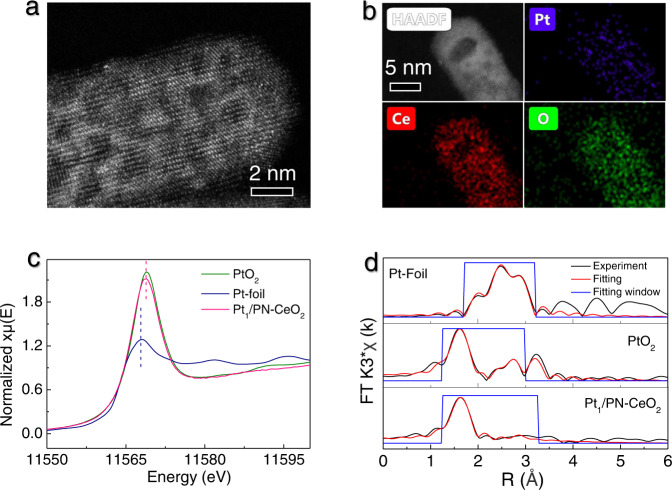
Characterizations of the Pt_1_/PN-CeO_2_ catalyst. **a** HAADF-STEM image and **b** EDS mapping. **c** XANES spectra of Pt-foil, PtO_2_, and Pt_1_/PN-CeO_2_. **d** The *k*^*3*^-weighted Fourier-transformed spectra derived from the EXAFS spectra of the Pt_1_/PN-CeO_2_ catalysts and PtO_2_.

Then, the single-atom Pt anchored on PN-CeO_2_ (Pt_1_/PN-CeO_2_) with 0.36 wt.% loading was successfully synthesized through a photo-assisted deposition process due to the strong trapping of metal species on the defective sites of PN-CeO_2_ from the DFT calculations (Supplementary Fig. 3). Both the specific surface area/pore structure (Supplementary Fig. 6) and levels of surface oxygen defects (Supplementary Fig. 7) of PN-CeO_2_ were preserved during the photo-assisted Pt deposition process, indicating the maintained surface FLP sites. According to the aberration-corrected high-angle annular dark-field scanning transmission electron microscopy (HAADF-STEM) image (Fig. 2a), the single-atom Pt in catalysts was experimentally demonstrated, which was further verified from the uniform Pt distribution on PN-CeO_2_ by the energy dispersive spectroscopy (EDS) mapping (Fig. 2b). X-ray absorption near edge structures (XANES) of Pt K-edge revealed that the white line peak of the Pt_1_/PN-CeO_2_ catalysts located at 11,568.7 eV (Fig. 2c), which was very close to that of PtO_2_. The *k*^*3*^-weight Fourier transforms of extended X-ray absorption fine structure (EXAFS) spectra of Pt_1_/PN-CeO_2_ delivered one prominent peak at ~1.63 Å, which was labeled as Pt–O bond (Fig. 2d). Also, the lack of Pt–Pt coordination again suggested no Pt particles and clusters in Pt_1_/PN-CeO_2_ (Fig. 2d and Table 1), indicating the atomically dispersed Pt supported on PN-CeO_2_.

**Table 1:** FT-EXAFS spectra and the fitting curves (without phase correction)

Sample	Path	*N*	Sigma^2^ (×10^−3^)	R (Å)	R-factor
Pt-foil	Pt–Pt	12 (set)^a^	4.71 ± 0.33	2.76 ± 0.01	0.002
PtO_2_	Pt–O	6.1 ± 1.2	3.3 ± 4.9	2.06 ± 0.07	0.008
	Pt–Pt	9.2 ± 2.8	5.6 ± 6.5	3.10 ± 0.84	
	Pt–O 2nd shell	11.9 ± 5.3	4.2 ± 7.2	3.48 ± 0.13	
Pt_1_/PN-CeO_2_	Pt–O	5.8 ± 0.6	3.0 ± 1.4	2.01 ± 0.60	0.015
	Pt–Pt	0	0	0	

^a^amp = 0.80 ± 0.04.

Then, the dispersion and chemical environments of Pt on PN-CeO_2_ were studied by diffuse-reflectance infrared Fourier-transform spectroscopy (DRIFTS). The peak at ~2090 cm^−1^ was assigned to the linearly adsorbed CO on isolated ionic Pt^2+^ (Supplementary Fig. 8)^38–40^, revealing that the Pt active sites existed as atomic dispersion on the surface of PN-CeO_2_ and coordinated with O atoms. Specifically, the two apparent peaks at 2099 and 2076 cm^−1^ could be attributed to the single-atom Pt located in the configurations of the type **I** and type **II** (Fig. 1d), respectively, owing to the lower valence state of Pt on oxygen vacancy adjacent to the FLP sites than it on oxygen vacancy in the distance (Supplementary Fig. 3). Therefore, combining with the HAADF-STEM, XANES, and DRIFTS results, the dual-active sites of single-atom Pt and FLPs were successfully constructed on the surface of Pt_1_/PN-CeO_2_ catalysts, where the single-atom Pt mainly occupied oxygen vacancy.

### Catalytic performance for H_2_ generation

**Fig. 3:**
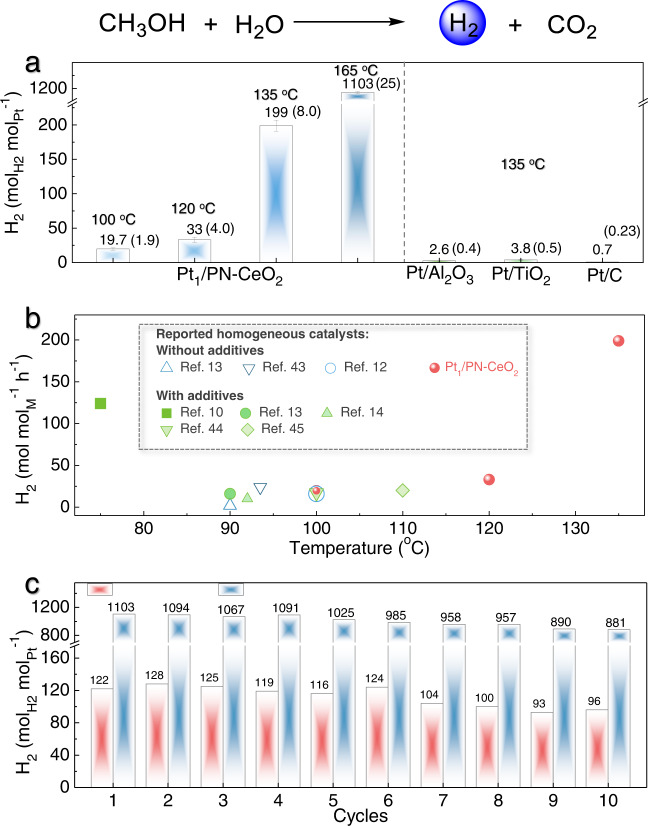
Catalytic performance. **a** H_2_ generation from methanol and H_2_O catalyzed by various Pt catalysts. The error is derived from three parallel experiments. Reaction conditions: catalysts (50 mg), CH_3_OH (40 mL), H_2_O (18 mL), n(CH_3_OH):n(H_2_O) = 1:1, and N_2_ (0.4 MPa). **b** Summary of the H_2_ generation catalyzed by Pt_1_/PN-CeO_2_ in the absence of additives compared with various reported homogeneous catalysts with or without additives at low temperature. **c** Cycling of Pt_1_/PN-CeO_2_ for H_2_ generation from methanol and H_2_O. The reaction time of each cycle was 1 and 3 h at 160 and 120 °C, respectively. Reaction conditions: Pt_1_/PN-CeO_2_ (50 mg), CH_3_OH (40 mL), H_2_O (18 mL), n(CH_3_OH):n(H_2_O) = 1:1, and N_2_ (0.4 MPa).

Then, the catalytic H_2_ generation of Pt_1_/PN-CeO_2_ through APRM methodology was evaluated under base-free conditions at various temperatures. To highlight the advantages of Pt_1_/PN-CeO_2_, Pt nanoparticles anchored on Al_2_O_3_, TiO_2_, and C with a loading of 0.5 wt.% were also prepared as the reference catalysts by impregnation method (Supplementary Fig. 9). At the reaction temperature of 135 °C, the Pt_1_/PN-CeO_2_ catalysts yielded a H_2_ generation rate of 199 mol_H2_ mol_Pt_^−1^ h^−1^, which was two or three orders of magnitude enhancement in the H_2_ generation rates catalyzed by Pt/Al_2_O_3_ (2.6 mol_H2_ mol_Pt_^−1^ h^−1^), Pt/TiO_2_ (3.8 mol_H2_ mol_Pt_^−1^ h^−1^), and Pt/C catalysts (0.7 mol_H2_ mol_Pt_^−1^ h^−1^) under the identical conditions, respectively (Fig. 3a). Even when the reaction temperature was reduced to 120 °C, the Pt_1_/PN-CeO_2_ catalysts still delivered a high H_2_ generation rate of 33 mol_H2_ mol_Pt_^−1^ h^−1^ (Fig. 3a). Comparatively, bare H_2_ was detected at this temperature for Pt catalysts anchored on other functional supports herein as well as in the previous reports^15,16,41,42^. In addition, the H_2_ generation of 19.7 mol_H2_ mol_Pt_^−1^ h^−1^ could be obtained by the fixed bed at a temperature as low as 100 °C. Most importantly, such a H_2_ generation rate catalyzed by Pt_1_/PN-CeO_2_ is comparative to and even better than the H_2_ generation rates when noble metal homogeneous catalysts are used in the presence of a high concentration strong base as additives at similar reaction temperatures (Fig. 3b and Supplementary Table 2)^12,13,43–45^. To the best of our knowledge, it is among one of the lowest temperatures for all previously reported heterogeneous metal catalysts to achieve the efficient H_2_ generation of reforming of methanol and water in the absence of any additives.

Most impressively, the selectivity of CO by-product catalyzed by Pt_1_/PN-CeO_2_ was only 0.032% and 0.027% at the reaction temperatures of 135 and 120 °C, respectively. When the reaction temperature was further increased to 165 °C, the H_2_ generation rate was significantly enhanced to 1103 mol_H2_ mol_Pt_^−1^ h^−1^. Encouragingly, the selectivity of CO was still maintained as low as 0.045% at this high reaction rate. Therefore, the Pt_1_/PN-CeO_2_ catalysts exhibited excellent catalytic performance for H_2_ generation from methanol and water, especially at low reaction temperatures.

In addition, the catalytic stability of Pt_1_/PN-CeO_2_, a critical factor of heterogeneous catalysts, was also evaluated through a cycling test at 165 °C (1 h per cycle) and 120 °C (3 h per cycle), respectively. At 165 °C, the H_2_ generation was slightly decreased from 1103 mol_H2_ mol_Pt_^−1^ to 881 mol_H2_ mol_Pt_^−1^ from the first cycle to the tenth cycle with a low CO selectivity <0.05% for each cycle (Fig. 3c). Also, the H_2_ generation was well maintained at the range of 93–128 mol_H2_ mol_Pt_^−1^ with a low CO selectivity <0.03% during 10 cycles at 120 °C (Fig. 3c). The preserved morphological features of the spent Pt_1_/PN-CeO_2_ catalysts could further reveal the satisfactory structure stability operated at both 165 °C and 120 °C (Supplementary Fig. 10a, b). Meanwhile, the surface Ce^3+^ fraction along with the Ce^3+^-O fraction of the used Pt_1_/PN-CeO_2_ catalysts, was similar to those of as-synthesized Pt_1_/PN-CeO_2_ (Supplementary Fig. 11), revealing that the FLP sites were stable throughout the catalytic hydrogen generation. After careful analysis of the HAADF-STEM images (Supplementary Fig. 10c), the Pt nanoclusters with a small amount were observed on the surface of the used Pt_1_/PN-CeO_2_ catalysts at 165 °C. A previous report has proved that the perimeter Pt active sites in the Pt-CeO_2_ catalytic system remain dynamically mobile for the reforming of *CO^46^. Thus, the decrease in H_2_ generation rate of Pt_1_/PN-CeO_2_ could be attributed to the slightly increased size of Pt active sites owing to the possible mobility of the atomically dispersed Pt on the surface of catalysts. Nevertheless, combining the base-free and absence of other additives, the high H_2_ generation rate and low CO selectivity, as well as the satisfactory long-term stability, make Pt_1_/PN-CeO_2_ featured as easy operation and high sustainability, exhibiting its great promises for practical H_2_ generation from APRM at low temperatures.

### Mechanism analysis

To verify our design concept of the Pt_1_-FLP dual-active sites for the enhanced activity and suppressed CO generation, we performed a series of control experiments to explore the catalytic mechanism of Pt_1_/PN-CeO_2_ for H_2_ generation *via* APRM process. As predicted from theoretical calculations, the single-atom Pt and FLPs on CeO_2_ can significantly activate CH_3_OH and H_2_O, respectively. Thereby, the contributions of Pt and FLPs for catalytic reaction were experimentally examined. In the absence of Pt, no H_2_ was generated from the reaction system with PN-CeO_2_ at 120 °C or 165 °C (Supplementary Table 2, Entry 8–9), demonstrating the inert nature of FLPs as well as the critical roles of Pt for APRM herein.

Then, to further inquiry the contributions of the FLP sites on PN-CeO_2_, the nonporous nanorods of CeO_2_ (NR-CeO_2_) and nanoparticles of CeO_2_ (NP-CeO_2_) were also selected as the referenced supports for H_2_ generation. We have previously demonstrated that the highly defective PN-CeO_2_ indexed by a large surface Ce^3+^ fraction exhibits a high probability to construct the interfacial FLP sites^32,47^. Thus, NR-CeO_2_ with a lower surface Ce^3+^ fraction (19.7%, Supplementary Fig. 12) exhibited less amount of surface FLP sites, in comparison with PN-CeO_2_ (30.8%, Supplementary Fig. 7). Due to the mismatched spatial configurations and large formation energy of oxygen vacancy, NP-CeO_2_ with the mainly exposed CeO_2_(111) surface as well as the lowest surface Ce^3+^ fraction (15.8%, Supplementary Fig. 13) exhibited the unsuccessful formation of interfacial FLP sites, as illustrated in our previous report^32^.

The Pt nanoparticles supported on PN-CeO_2_ (Pt/PN-CeO_2_), NR-CeO_2_ (Pt/NR-CeO_2_), and NP-CeO_2_ (Pt/NP-CeO_2_) were prepared by impregnation method with a Pt-loading of 0.51 wt.%, 0.52 wt.%, and 0.51 wt.%, respectively. The similar Pt sizes of Pt/PN-CeO_2_ (1.39 ± 0.31 nm), Pt/NR-CeO_2_ (1.36 ± 0.41 nm), and Pt/NP-CeO_2_ (1.31 ± 0.1 nm) excluded the size effects on H_2_ generation (Supplementary Fig. 14). Meanwhile, the surface Ce^3+^ fractions of Pt/PN-CeO_2_ (Supplementary Fig. 7), Pt/NR-CeO_2_ (Supplementary Fig. 12) and Pt/NP-CeO_2_ (Supplementary Fig. 13) were similar to their corresponding supports, indicating that the amount of FLP sites on various catalysts were preserved during the impregnation process. Therefore, the Pt/PN-CeO_2_, Pt/NR-CeO_2_, and Pt/NP-CeO_2_ catalysts exhibited the structural features of a similar Pt size, but various densities of FLPs in the order of Pt/PN-CeO_2_ > Pt/NR-CeO_2_ ≫ Pt/NP-CeO_2_.

**Fig. 4:**
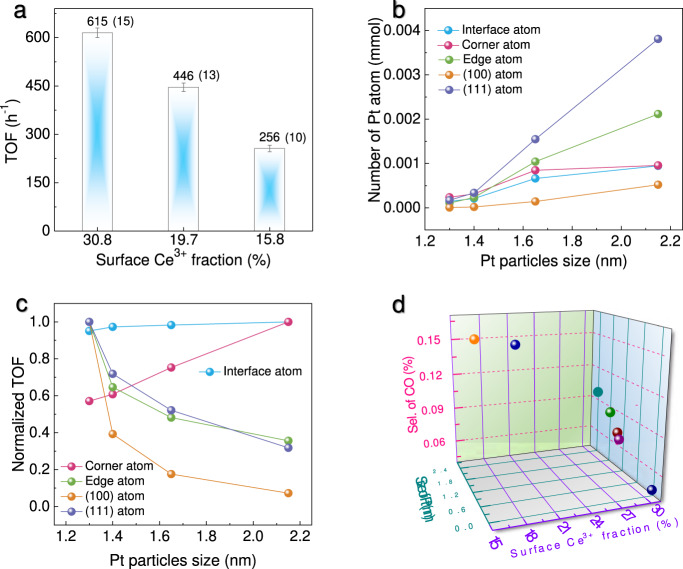
The influence of dual-active sites on the H_2_ generation performance. **a** Plots of TOF with surface Ce^3+^ fraction. The error is derived from three parallel experiments. **b** Plots of number of surface atoms per mole of Pt with Pt particle size of truncated cuboctahedron. **c** Plots of the normalized TOF values with Pt particle size. **d** The influence of Pt size and surface Ce^3+^ fraction on the selectivity of CO.

Among three catalysts, Pt/PN-CeO_2_, with the most abundant surface FLP sites, exhibited the highest apparent catalytic activity for H_2_ generation under the same reaction conditions (Supplementary Table 2, Entries 10–12), where the reaction temperature was at 165 °C. To further identify the intrinsic activity, the turnover frequency (TOF) values based on each exposed Pt atom were calculated to eliminate the influence of Pt sizes on catalytic activity. As shown in Fig. 4a, the TOF values increased significantly with the increase of the surface Ce^3+^ fractions (the indicator of the surface FLPs density). Specifically, the Pt/PN-CeO_2_ catalysts yielded a TOF of 615 h^−1^, which was 1.7 and 2.4 times higher than that of Pt/NR-CeO_2_ (364 h^−1^) and Pt/NP-CeO_2_ (256 h^−1^), respectively. Therefore, the FLPs sites can greatly accelerate the APRM process.

Based on the above-controlled experiments, the excellent catalytic performance of Pt_1_/PN-CeO_2_ for H_2_ generation *via* APRM could be attributed to cooperation between single-atom Pt and FLPs. As shown in theoretical calculations (Supplementary Fig. 4), the generated *CO on single-atom Pt undergoes a reforming with the formed *OH active species via water dissociation on FLP sites. Thus, only the interfacial Pt atom in nanoparticles may play the dominated contribution to achieve the synergistically cooperative catalytic effect for APRM. To examine this assumption, a series of Pt/PN-CeO_2_ with various Pt nanoparticles sizes were prepared by the impregnation method to obtain a various number of interfacial Pt atoms on PN-CeO_2_ surface. As shown in Supplementary Figs. 15 and 16, the sizes of Pt nanoparticles were increased from 1.30 ± 0.29 nm, to 1.39 ± 0.31 nm, and then to 1.65 ± 0.44 nm and finally to 2.15 ± 0.75 nm, when the loadings of Pt were controlled at 0.32, 0.51, 2.1, and 5.1 wt.%, respectively. Generally, the small Pt nanoparticles (<5.0 nm) are prone to display a morphology of truncated octahedron with the exposed (100) and (111) facets^48,49^. Then, the number of interface, corner, edge, (111), or (100) Pt atoms over the differently sized Pt anchored on PN-CeO_2_ were calculated and shown in Fig. 4b (the detailed analysis methodology in the Supplementary Information, including Supplementary Tables 4–7, and Supplementary Fig. 17).

Experimentally, the initial H_2_ generation rates for those catalysts were tested at 165 °C and summarized in Supplementary Table 2 (Entries 10 and 13–15). Assuming the uniform activity of each type of Pt site regardless of their sizes, the dominated active Pt species should show a near linearly increased activity as well as a constant catalytic activity (indexed by TOF) for the H_2_ generation. Then, the TOF value of each type of Pt active site was calculated (Supplementary Fig. 18) and normalized to the maximum one of each type of Pt active site. As shown in Fig. 4c, the normalized TOF values of the corner Pt increased continuously with the increased sizes of Pt particles. While, the normalized TOF values of the edge, (110), and (111) Pt decreased monotonically with the increased Pt sizes. Thus, none of them were considered the real active sites for APMR. Surprisingly, the normalized TOF values of the interface Pt were almost constant regardless of their sizes (Fig. 4c), suggesting that the interface Pt atoms were the dominated active sites for the H_2_ generation through the reforming of methanol and water.

Beyond activity, the release of CO during H_2_ generation was greatly suppressed in the presence of abundant FLPs sites. The FLP sites on PN-CeO_2_ effectively promote the H_2_O activation for the generation of *OH species (Fig. 1), which can further improve the reforming of *CO to CO_2_ and thereby reduce the direct release of CO. Consistently, the CO selectivity reduced experimentally with the increased surface Ce^3+^ fractions (an indicator of the number of FLPs, Fig. 4d). Importantly, the CO generation was also largely suppressed when the sizes of Pt were reduced (Fig. 4d). The generated *OH intermediates on CeO_2_ are easily accessible to directly attack the *CO adsorbed on the interfacial Pt atoms, also resulting in the promoted reforming of *CO to CO_2_. Therefore, the co-existence of the interfacial Pt and FLPs effectively improves the reforming of *CO to suppress the CO generation.

From the above analysis, the cooperation of interfacial Pt atoms and FLP sites is experimentally and theoretically demonstrated for the selective H_2_ generation from methanol and H_2_O at low temperatures. The Pt_1_/PN-CeO_2_ catalysts with single-atom Pt and abundant surface oxygen defects fully expose the Pt atom and construct the richest interfacial Pt_1_-FLP dual-active sites, thereafter achieving the significant promotion of H_2_ production with satisfactory CO suppression for APRM at low temperatures.

In order to further clarify the essence of dual-active sites for each step to boost the catalytic performance of Pt_1_/PN-CeO_2_, the H/D isotope experiments were performed on the Pt_1_/PN-CeO_2_, Pt/PN-CeO_2_, and Pt/NR-CeO_2_ catalysts under the given reaction conditions. The Pt_1_/PN-CeO_2_ and Pt/PN-CeO_2_ catalysts exhibited the close density of FLP sites but different amounts of interface Pt atom. While Pt/PN-CeO_2_ and Pt/NR-CeO_2_ with similar Pt sizes showed different densities of the interfacial FLP sites. Derived from their H_2_ generation rates (Supplementary Fig. 19), the value of *k*_H2O_/*k*_D2O_ for Pt/PN-CeO_2_ (2.2) was slightly lower than that for Pt/NR-CeO_2_ (2.6). Due to the small fraction of interfacial Pt atoms, the promotion of FLPs sites on PN-CeO_2_ could be vaguely embodied by the nanoscaled catalysts. When the metal sites are all interfacial Pt atoms as featured in Pt_1_/PN-CeO_2_, the promotion by FLPs for H_2_O activation was significantly enhanced, as revealed from the lowest value of *k*_H2O_/*k*_D2O_ (1.5). This phenomenon further indicated that the interfacial Pt atoms were the dominated active sites for the H_2_ generation *via* the accelerated reforming of *CO and *OH.

**Fig. 5:**
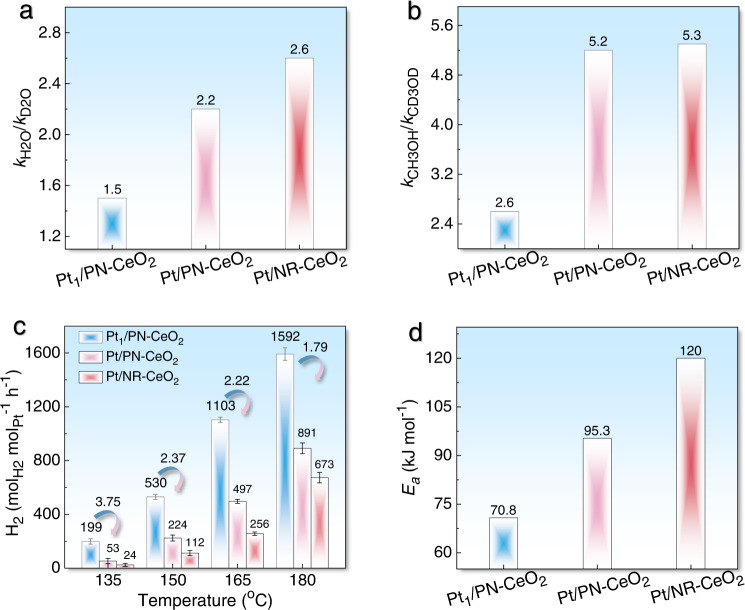
Kinetic analysis of dual-active sites for H_2_ generation from methanol and H_2_O. **a** Ratios of H_2_ generation rate from CH_3_OH/H_2_O to CH_3_OH/D_2_O. **b** Ratios of H_2_ generation rate from CH_3_OH/H_2_O to CD_3_OD/H_2_O. **c** H_2_ generation rates at various reaction temperatures catalyzed by Pt_1_/PN-CeO_2_, Pt/PN-CeO_2_, and Pt/NR-CeO_2_. The error is derived from three parallel experiments. **d** *E*_*a*_ of various Pt catalysts for H_2_ generation from methanol and H_2_O.

Besides, the Pt/PN-CeO_2_ and Pt/NR-CeO_2_ catalysts exhibited similar values of *k*_CH3OH_/*k*_CD3OD_ (~5.2, Fig. 5b), predicating the CH_3_OH activation on the nanoscaled Pt particles and the negligible effects of the CeO_2_ supports. Comparatively, the significantly decreased *k*_CH3OH_/*k*_CD3OD_ value (2.6) for Pt_1_/PN-CeO_2_ indicated the single-atom Pt benefits the activation of CH_3_OH (Fig. 5b). Therefore, isotope experiments strongly suggest that the dual-active sites of single-atom Pt and FLPs are kinetically beneficial for the simultaneously promoted activation of CH_3_OH.

In comparison with Pt/PN-CeO_2_ and Pt/NR-CeO_2_, the Pt_1_/PN-CeO_2_ catalysts exhibited the highest H_2_ generation rate at each reaction temperature (Fig. 5c). More importantly, the ratios of H_2_ generation rates of Pt_1_/PN-CeO_2_ to Pt/PN-CeO_2_ increased gradually from 1.79, to 2.22, and then to 2.37, and final to 3.75 at reaction temperatures of 180, 165, 150, and 135 °C, respectively (Fig. 5c), demonstrating the highest capability of Pt_1_/PN-CeO_2_ for H_2_ generation, especially at low temperatures. Also, the derived activation energy (*E*_*a*_) of Pt_1_/PN-CeO_2_ for H_2_ generation via APMR reaction was 70.8 kJ mol^−1^, which was 1.4 and 1.7 times lower than the *E*_*a*_ of Pt/PN-CeO_2_ (95.3 kJ mol^−1^) and Pt/NR-CeO_2_ (120.0 kJ mol^−1^), respectively (Fig. 5d and Supplementary Fig. 20). Therefore, the improved activation of H_2_O and CH_3_OH results in the decreased energy barrier of H_2_ generation, further leading to the remarkable catalytic performance of Pt_1_/PN-CeO_2_ at low temperatures.

Experimentally, the single-atom Pt sites for the effective CH_3_OH activation have been proved by the obviously decreased value of k_CH3OH_/k_CD3OD_ for Pt_1_/PN-CeO_2_. In order to gain deep insights, DFT calculations were also employed to understand the activation of CH_3_OH on the constructed Pt_1_-FLP dual-active sites (Fig. 1d). Three catalytic models of Pt (111), Pt_1_/CeO_2_(110), and Pt_1_/CeO_2_(110)-FLP were also used to represent the Pt nanoparticles, single-atom Pt on oxygen vacancy of CeO_2_(110) and single-atom Pt on FLP site of CeO_2_(110), respectively. As shown in Supplementary Fig. 21, the largest energy barrier of 0.75 eV for CH_3_OH decomposition on Pt(111), is the first step of CH_3_OH transformation into *CH_3_O and *H, consistent with literature^11^. For Pt_1_/CeO_2_(110), the step of *CH_3_O transformation into *CH_2_O and *H exhibits a similar energy barrier (0.8 eV) with it on Pt(111) surface. These results revealed that the size of Pt was not the critical factor for CH_3_OH dissociation. However, the energy barrier for the CH_3_OH decomposition is significantly reduced to only 0.23 eV on Pt_1_/CeO_2_(110)-FLP along with the transformation of *CHO into *CO and *H (Supplementary Fig. 21). In this configuration, the Lewis acidic Ce^3+^ as a co-active site interacts with CH_3_OH molecule, further promoting the CH_3_OH dissociation afterward. Therefore, the presence of FLP sites adjacent to single-atom Pt results in the obvious reduced energy barrier of CH_3_OH dissociation.

To experimentally detect the roles of Lewis acidic Ce^3+^, the adsorption behavior of methanol on the surface of Pt_1_/PN-CeO_2_ catalysts were investigated. As shown in Supplementary Fig. 22, methanol molecules could adsorb on the oxygen vacancy of PN-CeO_2_ and then dissociate to methoxy species owing to the presence of the bridged methoxy species with the characteristic peak at 1033 cm^−1^. After introducing single-atom Pt, another bridged methoxy species at 1058 cm^−1^ appeared, as revealed from the FTIR spectra. Compared with the adsorption behavior of methanol on PN-CeO_2_, this appeared methoxy species could be attributed to the bridged configuration of methoxy on the single-atom Pt and adjacent Ce^50^. Previous reports have proved that the CeO_2_ supports exhibited no catalytic ability for methanol dissociation^27^. Therefore, the bridged methoxy species on the single-atom Pt and adjacent Ce were detected as the critical intermediate for methanol dissociation on the Pt_1_/PN-CeO_2_ catalysts.

In addition, the d-band electron structures of various Pt sites were characterized by high-resolution valence-band XPS spectra. Due to the synergistic effects of the PN-CeO_2_ supports with abundant defect of oxygen vacancy and the atomic dispersion of Pt active sites, the Pt_1_/PN-CeO_2_ catalysts exhibited a significant shift of the d-band center towards the VBM (Supplementary Fig. 23), resulting in a downward shift of the antibonding states. According to the d-band center theory, the strong bonding of adsorbates occurs if the antibonding states are shifted up relative to the Fermi level, and weak bonding occurs if the antibonding states are shifted down^51,52^. Therefore, the Pt_1_/PN-CeO_2_ catalysts also exhibited the weakest bonding with CO_2_ molecule, facilitating its desorption form single-atom Pt active sites.

**Fig. 6:**
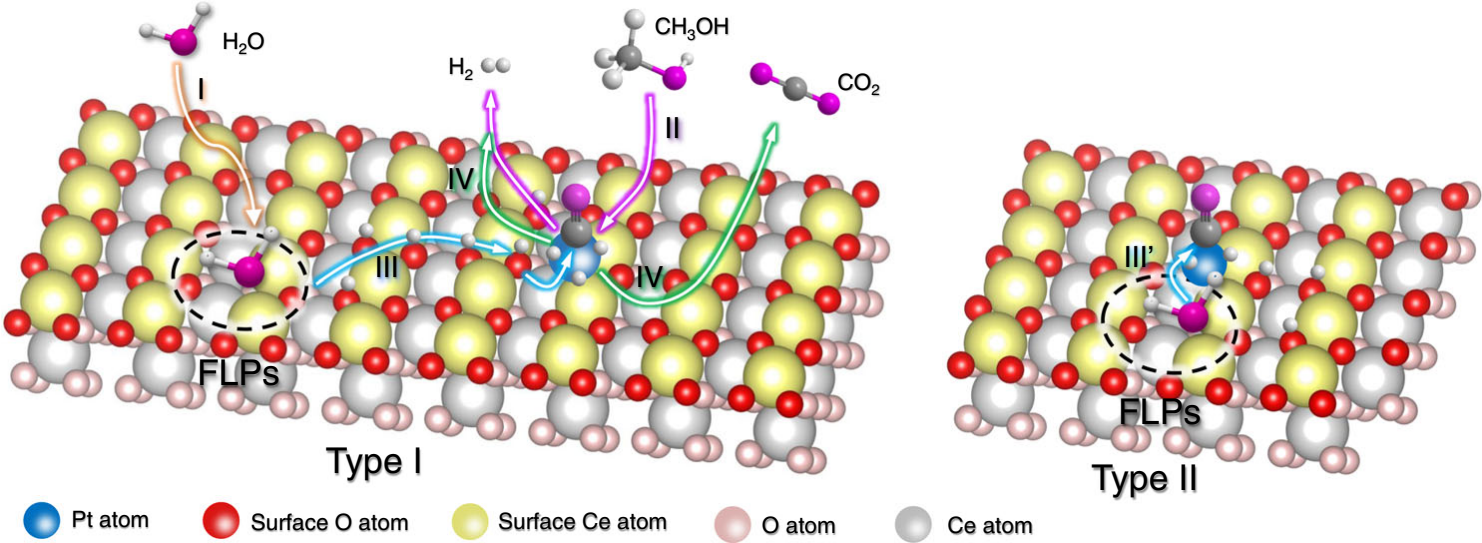
Proposed reaction process. Catalytic pathway for H_2_ generation from methanol and H_2_O catalyzed by the Pt_1_-FLP dual-active sites constructed on Pt_1_/PN-CeO_2_.

Based on the various control experiments, kinetic analysis, and DFT calculations, the constructed Pt_1_-FLP dual-active site in Pt_1_/PN-CeO_2_ boosted a high catalytic performance for H_2_ generation through APMR at low temperatures. A proposed catalytic process is illustrated in Fig. 6. (**I**) H_2_O molecule is adsorbed and dissociated into *H and *OH species on the FLP sites. (**II**) Methanol molecule is easily dissociated into *H and *CO on the single-atom Pt with the help of the Lewis acidic Ce^3+^ site of FLP sites or the nearly Ce^3+^ site, releasing H_2_ molecule in methanol. For the type **I** of Pt_1_-FLP dual-active sites (Fig. 6a), due to the long spatial distance between Pt and FLPs, (**III**) the *H and *OH species would diffuse on the surface of CeO_2_ from FLP sites to the single-atom Pt sites, and then reform of *CO intermediates on the interfacial Pt active sites to give CO_2_ along with the production of extra H_2_. (**IV**) The generated H_2_ and CO_2_ molecules release from the surface of catalysts. Meanwhile, for the type **II** of Pt_1_-FLP dual-active sites (Fig. 6b), (**III**), with the help of the abundant *OH species on FLP sites, the reforming of *CO intermediate effectively occurs on the adjacent Pt active sites without the diffusion of the *H and *OH species. Other steps on the type **II** of Pt_1_-FLP dual-active sites are similar to those on the type **I** of Pt_1_-FLP dual-active sites. Therefore, the Pt_1_/PN-CeO_2_ catalysts with the richest interface between Pt and PN-CeO_2_ and abundant FLP sites enable the efficient CH_3_OH and H_2_O dissociation and effectively reformed the *OH and *CO intermediate, facilitating the H_2_ production at low temperatures.

## Discussion

The dual-active site catalyst that comprises the atomically dispersed Pt and FLPs on PN-CeO_2_ has been successfully developed for the low-temperature H_2_ generation with the suppressed CO from methanol and H_2_O. Methanol is efficiently dissociated into *H and *CO intermediates with the help of Lewis acidic Ce^3+^ sites of FLPs, in which the energy barrier is only 0.23 eV. The FLP sites constructed on PN-CeO_2_ enable the kinetically and dynamically favorable H_2_O dissociation, producing abundant surface hydroxyls for the subsequent transformation of *CO and *OH into *CO_2_ and *H. Therefore, the H_2_ generation from methanol and H_2_O at low temperatures is significantly accelerated between single-atom Pt and FLP sites. Due to hydrogen with the low CO concentration required of fuel cells as well as other applications, herein, the catalytically generated hydrogen with a CO concentration of 270 ppm from methanol and H_2_O by Pt_1_/PN-CeO_2_ has to be handled carefully and purified as the power supplies of the fuel cell at this stage. Nevertheless, this new catalyst featured with the facile synthesis and high activity as well as the suppressed CO generation at low temperatures still paves a possible way towards a commercially achievable liquid sunshine roadmap.

## Methods

### Preparation of PN-CeO_2_

The PN-CeO_2_ supports were prepared by a two-step hydrothermal process at various temperatures^47,53^. Initially, aqueous solutions of Ce(NO_3_)_3_·6H_2_O (1.736 g in 10 mL of deionized water) and NaOH (19.2 g in 70 mL of deionized water) were mixed slowly in a Pyrex bottle (100 mL) and reacted for 0.5 h at room temperature under continuous stirring. After aging for another 1 h, the reaction was continued at 100 °C for 24 h for the first hydrothermal process. Then, the reaction mixture was cooled naturally to room temperature. The CeO_2_/Ce(OH)_3_ solids were collected by centrifugation, intermittently washed with deionized water and ethanol for three times, and dried in air at 60 °C. After that, the secondary hydrothermal process was performed to treat the CeO_2_/Ce(OH)_3_ precursors with 2 mg mL^−1^ at 100 °C for 12 h. Finally, the PN-CeO_2_ supports were obtained after centrifugation and dried in air at 60 °C.

### Preparation of the Pt_1_/PN-CeO_2_ catalysts

The Pt_1_/PN-CeO_2_ catalysts were synthesized through a photo-assisted deposition process. Initially, 300 mg of PN-CeO_2_ supports were dispersed in 40 mL of 6 vol.% methanol aqueous solution. After adding the desired amount of H_2_PtCl_6_, the mixture was irradiated under Xe lighter for 3 h. Then, the products were collected by centrifugation to remove the free H_2_PtCl_6_. Finally, the Pt_1_/PN-CeO_2_ catalysts were reduced by 5 vol.% H_2_/Ar at 300 °C for 2 h.

### Characterizations

The catalysts were characterized by a Shimadzu X-ray diffractometer (Model 6000) using Cu Kα radiation. TEM studies were conducted on the Hitachi HT-7700 with an accelerating voltage of 120 kV. High-resolution and dark-field TEM images were acquired from the Tecnai G2 F20 S-twin transmission electron microscope at 200 kV. The surface area was measured by N_2_ physisorption (Micromeritics, ASAP 2020 HD88) based on Brunauer-Emmet-Teller (BET) method. XPS were acquired using a Thermo Electron Model K-Alpha with Al K_α_ as the excitation source.

### Catalytic performance for hydrogen generation from methanol and H_2_O

For a typical catalytic reaction, 40 mL of methanol and 18 mL of H_2_O with 50 mg catalysts were mixed in a 500 mL autoclave equipped with a temperature controller and a pressure detector. The temperature of the reaction system quickly increased to the given temperature within 10 min. After the reaction, the mixture was collected in a 1 L of gas sampling bag and then analyzed by GC with TCD and FID detector.

## Supplementary information

Supplementary information

Peer Review File

## Data Availability

The authors declare that the main data supporting the findings of this are available within the article and supplementary information from the corresponding author upon reasonable request. The main data generated in this study are provided in the Source data file. Source data are provided with this paper.

## Source data

Source Data
