# Supplementary material for: Sustainable production of hydrogen with high purity from methanol and water at low temperatures
Source: Nat Commun. 2022 Sep 21;13:5527. doi: 10.1038/s41467-022-33186-z (PMC9492729; doi:10.1038/s41467-022-33186-z)
Supplement: Supplementary file 1 — Supplementary information [file 41467_2022_33186_MOESM1_ESM.pdf]

Sustainable production of hydrogen with high purity from methanol and water at low temperatures

Sai Zhang[1,2], Yuxuan Liu[3], Mingkai Zhang[3], Yuanyuan Ma[1], Jun Hu[4,*] and Yongquan Qu[1,*]

[1] *School of Chemistry and Chemical Engineering, Northwestern Polytechnical University, Xi'an, 710072, China*

[2] *Research & Development Institute of Northwestern Polytechnical University in Shenzhen, Shenzhen, 518057, China*

[3] *Center for Applied Chemical Research, Frontier Institute of Science and Technology, Xi'an Jiaotong University, Xi'an, 710049, China.*

[4] *School of Chemical Engineering, Northwest University, Xi'an, 710069, China.*

Correspondence and requests for materials should be addressed to Hu J. and Qu Y. Q. (Email: hujun@nwu.edu.cn and yongquan@nwpu.edu.cn)

Supplementary Methods.

Preparation of the nanorods of CeO_2 (NR-CeO_2) supports. The NR-CeO_2 supports were prepared by traditional hydrothermal process. Initially, aqueous solutions of $Ce(NO_3)_3 \cdot 6H_2O$ (1.736 g in 10 mL of deionized water) was added into the NaOH (19.2 g in 70 mL of deionized water) solutions for 0.5 h at room temperature by continuous stirring. After aging for another 1 h, the reaction was continued at 160 °C for 12 h. Finally, the reaction mixture was then cooled naturally to room temperature and intermittently washed with deionized water and ethanol for three times.

Preparation of nanoparticles of CeO_2 (NP-CeO_2). The NP-CeO_2 supports were obtained by calcination $Ce(NO_3)_3 \cdot 6H_2O$ at 500 °C for 2 h.

Preparation of the Pt/PN-CeO_2 catalysts. Pt nanoparticles supported on the surface of PN-CeO_2 were obtained by traditional impregnation method. Initially, 300 mg PN-CeO_2 supports were dispersed in 10 mL ethanol. Then, 1 mL H_2PtCl_6 solution (Pt: 0.5 *wt.*%) was added in the mixture and stirred 1 h at room temperature. After that, the mixture was slowly heated to 90 °C to remove the ethanol. Finally, the Pt/PN-CeO_2 catalysts were reduced by 5 *vol.*% H_2/Ar at 300 °C for 2 h.

The Pt/NR-CeO_2 and Pt/NP-CeO_2 catalysts were obtained by the similar process only changing the PN-CeO_2 supports to the NR-CeO_2 or NP-CeO_2 supports.

Catalytic generation of hydrogen from methanol and H_2O. For a typical catalytic reaction, 40 mL methanol and 18 mL H_2O with 50 mg catalysts were mixed in a 500 mL autoclave with a temperature and pressure detector. The temperature of the reaction system quickly increased to the given temperature within 10 min. After

reaction, the mixture was collected by a 1 L gas sampling bag. The gas products were analyzed by GC with TCD and FID detector.

DFT calculation.

CASTEP module of the Materials Studio software (Accelrys Inc.) was employed for the quantum chemistry calculations. Self-consistent periodic Density Functional Theory (DFT) was adopted to explore the electronic structure and catalytic activities. Perdew-Burke-Ernzerhof (PBE) approximation was selected as the Generalized Gradient Approximation (GGA) method to calculate the exchange-correlation energy.[1-3] The Broyden-Fletcher-Goldfarb-Shanno (BFGS) scheme was selected as the minimization algorithm.[3] And ionic cores were represented by an ultrasoft pseudopotential in reciprocal space. CeO_2 (110) facets with 3×1 supercell was built from bulk CeO_2 (a=b=5.13 Å, c=3.64 Å, α=β=γ=90°) were used based on the XRD and TEM data. Vacuum region is set as 15 Å in order to neglect the electrostatic interactions between two sides of a slab. Based on the XPS results, one oxygen vacancy (Vo) and two Vo are generated on the CeO_2 (110) facets named as CeO_2 (110)+Vo and CeO_2 (110)+2Vo, respectively. The energy cutoff is 410 eV and the SCF tolerance is 0.5×10^{-7} eV/atom. And the k-points were set 1×2×1. The optimization is completed when the energy, maximum force, maximum stress and maximum displacement are smaller than 5.0×10^{-6} eV/atom, 0.01 eV/ Å, 0.02 GPa and 5.0×10^{-4} Å, respectively.

The vacancy formation energy (E_{Vo}) can be estimated by Eq. S1.[4]

$$E_{Vo}=E_{ds}+E_{o}-E_{ps} \qquad \text{(S1)}$$

Where E_{ds} is total energy of the defective surface; E_{ps} is total energy of perfect

surface and E_o is the half energy of an O_2 molecule in the gas phase; E_{o_2} is the energy of an O_2 molecule in the gas phase

The adsorption energy (E_{ads}) between a facet and adsorbed particles was computed by Eq. S2:

$$E_{ads} = E_{*ads} - E_{ads} - E_{*} \qquad (S2)$$

Where E_{*ads} is the total energy of the facet and adsorbent; E_{ads} is the energy of adsorption molecules; and E_{*} is the energy of facet. Zero-Point Energy (ZPE) correction were not considered during the above calculations.[5]

Based on previous experiments and simulations, a plausible pathway of WGS on CeO_2 (110) facet has been reported as the series of reactions 1 to 6 (R1 to R6), where the subscript of "*" represents the adsorbed species. Reaction free energy change is calculated for the energy of products plus reactions.

+CH_3OH=CH_3OH (R1)

CH_3OH=CH_3O+*H (R2)

*CH_3O+*H=*CH_2O+2*H (R3)

*CH_2O+2*H=*CHO+3*H (R4)

*CHO+2*H=*CO+4*H (R5)

*CO+4*H=CO+4*H (R6)

Supplementary Table 1. Summary of surface properties of various catalysts.

Sample	Ce^{3+} fraction (%)	Ce^{3+}-O fraction (%)
PN-CeO_2	30.8	47.1
Pt_1/PN-CeO_2	34.7	45.4
NR-CeO_2	19.7	34.5
Pt/NR-CeO_2	25.6	38.4
NP-CeO_2	15.8	23.7
Pt/NP-CeO_2	19.2	28.7
Pt_1/PN-CeO_2-used	32.7	47.3

Supplementary Table 2. Summary of H_2 generation from methanol and H_2O by various Pt catalysts

Entry	Catalysts	Pt loading (*wt.*%)	Temperature (°C)	H_2 generation rate ($mol_{H2}\ mol_{Pt}^{-1}\ h^{-1}$)	Sel. of CO (%)
1	Pt_1/PN-CeO_2	0.36	135	199	0.032
2	Pt/Al_2O_3	0.54		2.6	--
3	Pt/TiO_2	0.52		3.8	--
4	Pt/C	0.51		0.7	--
5	Pt_1/PN-CeO_2	0.36	165	1103	0.045
6			120	33	0.027
7			100	19.7	0.16
8	PN-CeO_2	0	165	0	--
9			120	0	--
10	Pt/PN-CeO_2	0.51	165	497	0.072
11	Pt/NR-CeO_2	0.52		256	0.153
12	Pt/NP-CeO_2	0.51		202	0.159
13	Pt/PN-CeO_2	0.32		589	0.067
14	Pt/PN-CeO_2	2.1		479	0.086
15	Pt/PN-CeO_2	5.1		246	0.098

Reaction condition: catalyst (50 mg), n(CH_3OH):n(H_2O)=1:1, 58 mL total volume of liquid, 165 °C and 1 h.

Supplementary Table 3. Summary of H_2 generation from methanol and H_2O catalyzed by various homogeneous catalytic system.

Entry	Catalysts	Additive	$n(CH_3OH)/n(H_2O)$	Temperature (°C)	H_2 (mmol $mmol_M^{-1}$ h^{-1})	Ref.
1	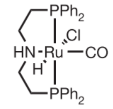	0.5 M NaOH	9:1	72	124	6
		8 M KOH	9:1	91	1023	
2	Ru/2-hydroxypyridine	1.2 Equiv.	1:1	110	20	7
3	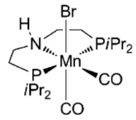 1	8 M KOH	9:1	92	10	8
4	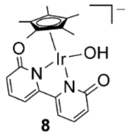 8	--	1:4	90	2	9
		1 mol% NaOH	1:4	90	16	
5	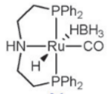	--	4:1	93.5	24	10
6	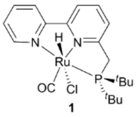 1	2 Equiv.	1:5	100-105	17	11
7	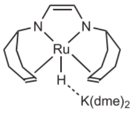	--	1:1	100	16	12
8	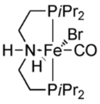	8 M KOH	--	91	702	13
9	Carbon dots/C_3N_4	--	--	80	19.5 μmol g^{-1} h^{-1}	14

The calculation of the number of atoms on the surface.

Supplementary Table 4. Formulas to calculate the atom numbers at different positions for each Pt particle as well as the number of Pt particles.

Total atom number of each particle (N_T)	$16m^3$-$33m^2$+$24m$-6
Surface atom number of each particle (N_S)	$30m^2$-$60m$+32
Corner atom number of each particle (N_{corner})	24
Edge atom number of each particle (N_{edge})	36(m-2)
100 atom number of each particle (N_{100})	$6(m-2)^2$
111 atom number of each particle (N_{111})	8($3m^2$-$9m$+7)
Number of Pt particles (N_P)	n_{Pt}×N_A/N_T

The diameter of Pt atom (d_{Pt} atom) is 0.276 nm. Between the particle size (d) and the number of atoms involved in one cuboctahedron shaped Pt particle follows the relationship of 1.105×$N_T^{1/3}$×d_{Pt} atom. That is the Equation 1:

$$d = 1.105 \times {N_T}^{1/3} \times d_{Pt} \quad (1)$$

Thus, total atom number of each particle (N_T) can be calculated by the Equation 2:

$$N_T = \left(\frac{d}{1.105 \times d_{Pt}}\right)^3 \quad (2)$$

The total number of Pt nanoparticles (N_P) in various Pt/PN-CeO_2 can be obtained by the Equation 3.

$$N_P = \left(\frac{m_C \times w_C}{M_{Pt}} \times 6.02 \times 10^{23}\right) / N_T \quad (3)$$

Where m_C is the quality of catalysts, w_C is the loading of Pt, M_{Pt} is the relative molecular mass of Pt, N_T is the total atom number of each particle.

Due to truncated cuboctahedron showing the half morphology of cuboctahedron (a highly symmetric atomic structure), the relative ratios of different site atoms for both shapes at the same particle size are the same. m is the number of atoms lying on an equivalent edge (corner atoms included).

The value of m of each catalyst can be calculated by the Equation 4.

$$N_T = 16 \times m^3 - 33 \times m^2 + 24 \times m - 6 \quad (4)$$

And, the m value of each Pt/PN-CeO_2 catalysts were summarized in the Table S2.

Then, the number of various active of each type were obtained and summarized in Table S3.

Meanwhile, the number of interface Pt atoms in a Pt nanoparticle could be obtained by the following process.

As shown in Supplementary Figure 17, the interface Pt atoms in the Pt/PN-CeO_2 catalysts are defined as the surface Pt atoms, which are direct contact with PN-CeO_2 supports. The interface Pt atoms can be considered as a circle. Thus, the diameter of the formed circle is the size of supported Pt nanoparticles. And the particles size and the number of interface atoms in a Pt nanoparticle follows the Equation 5:

$$\pi \times d = N_{interface} \times 2 \times d_{Pt} \quad (5)$$

Thus, the number of interface Pt atom were obtained and summarized in Table S3.

The total active sites of each type can be calculated by multiplying the number of nanoparticles (N_T) by the number of active sites on each particle, as shown in Table S4.

Supplementary Table 5. Summary of the N_T and *m* of each Pt/PN-CeO_2 catalyst.

Pd loading (*wt.* %)	Pd size (*d*)	N_T	*m*
0.3	1.3 ± 0.29	77.45	2.30
0.5	1.39 ± 0.31	96.73	2.49
2.2	1.65 ± 0.44	158.36	2.82
5.5	2.15 ± 0.75	350.35	3.48

Supplementary Table 6. Summary of the number active sites of each type

Pd size (*d*)	N_S	N_{corner}	N_{edge}	N_{100}	N_{111}	$N_{interface}$
1.3 ± 0.29	52.70	24	10.80	0.54	17.36	14.80
1.39 ± 0.31	68.96	24	17.78	1.46	25.71	15.93
1.65 ± 0.44	101.48	24	29.56	4.04	43.88	18.77
2.15 ± 0.75	185.77	24	53.10	13.06	95.62	23.73

Supplementary Table 7. Summary of the total number active sites of each type

Pd size (*d*)	mmol					
	N_S	N_{corner}	N_{edge}	N_{100}	N_{111}	$N_{interface}$
1.3 ± 0.29	0.000518	0.000236	0.000106	5.31×10^{-6}	0.000171	0.000145
1.39 ± 0.31	0.000905	0.000315	0.000233	5.31×10^{-5}	0.000337	0.000209
1.65 ± 0.44	0.00358	0.000846	0.00104	0.000143	0.00155	0.000662
2.15 ± 0.75	0.00740	0.000956	0.00211	0.00052	0.00381	0.000945

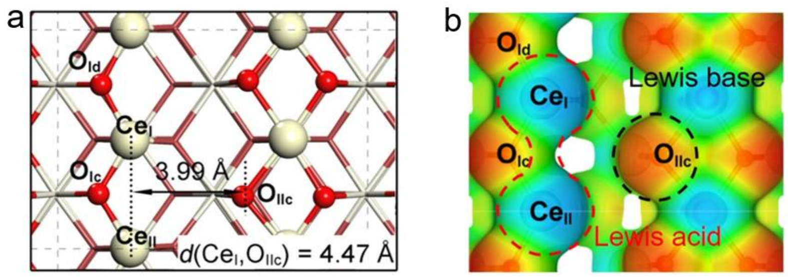

Supplementary Figure 1 | Optimized FLPs on CeO_2(110) surface. The (**a**) spatial configuration and (**b**) electron-density isosurface of the FLPs sites.

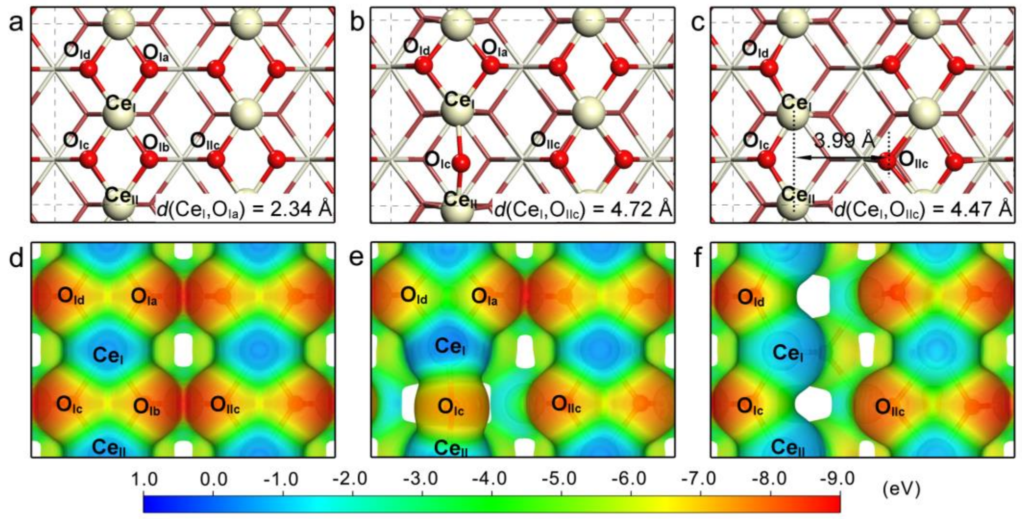

Supplementary Figure 2 | Schematic images of concept for design of solid frustrated Lewis pairs in CeO_2 crystal structure. The constructive process of FLPs sites on CeO_2 surface have been described in the previous reports (Solid frustrated-Lewis-pair catalysts constructed by regulations on surface defects of porous nanorods of CeO_2, Sai Zhang and Zhengqing Huang, *Nat. Commun.* 2017, *8*, 15266). (**a**) Optimized structure of ideal CeO_2(110). (**b)** Optimized structure of CeO_2(110) with one oxygen vacancy. (**c)** Optimized structure of CeO_2(110) with two adjacent oxygen vacancies. (**d**) Electron-density isosurface of ideal CeO_2(110). (**e**) Electron-density isosurface of CeO_2(110) with one oxygen vacancy. (**f)** Electron-density isosurface of

$CeO_2(110)$ with two oxygen vacancies. The electron-density isosurfaces are plotted at 0.01 e/bohr^3. The color bar represents the electrostatic potential scale.

Note:

For ideal $CeO_2(110)$ surface, surface Ce and O atoms form Lewis acid-base adjuncts (Figure S2a and S2d). When one O atom (O_{Ib}) is removed from $CeO_2(110)$ surface, the Lewis acidic Ce_I and Lewis basic O_{IIc} atoms are expected to construct FLP sites of Ce_I-O_{IIc} after structural relaxation (Figure S2b). However, the electronic interaction between Ce_I and O_{Ia}/O_{Ic} will still hinder the activation of small molecules on the Ce_I-O_{IIc} active sites (Figure S2e). Therefore, the weak FLP-like activation is obtained on traditional CeO_2 materials. When the second adjacent surface oxygen (O_{Ia}) is removed, the reduced Ce cations (Ce_I and Ce_{II}) and surface lattice oxygen (O_{IIc}) are independent Lewis acidic and basis sites (Figure S2c and S2f), respectively. Notably, two adjacent reduced surface Ce sites (Ce_I and Ce_{II}) and lattice O_{IIc} is constructed the FLPs site of (Ce_I,Ce_{II})-O_{IIc} with a shorter distance (3.99 Å).

However, the construction of surface FLPs cannot be realized by removing surface oxygen atoms on $CeO_2(111)$. Due to the low formation of oxygen vacancy on $CeO_2(100)$, the spatial configuration for the formation of FLPs sites is unstable. Therefore, $CeO_2(110)$ surface instead of $CeO_2(100)$ and $CeO_2(111)$ surfaces exhibits the highest possibility for FLPs construction owing to the unique FLPs configuration and formation energy of oxygen vacancies on various surfaces.

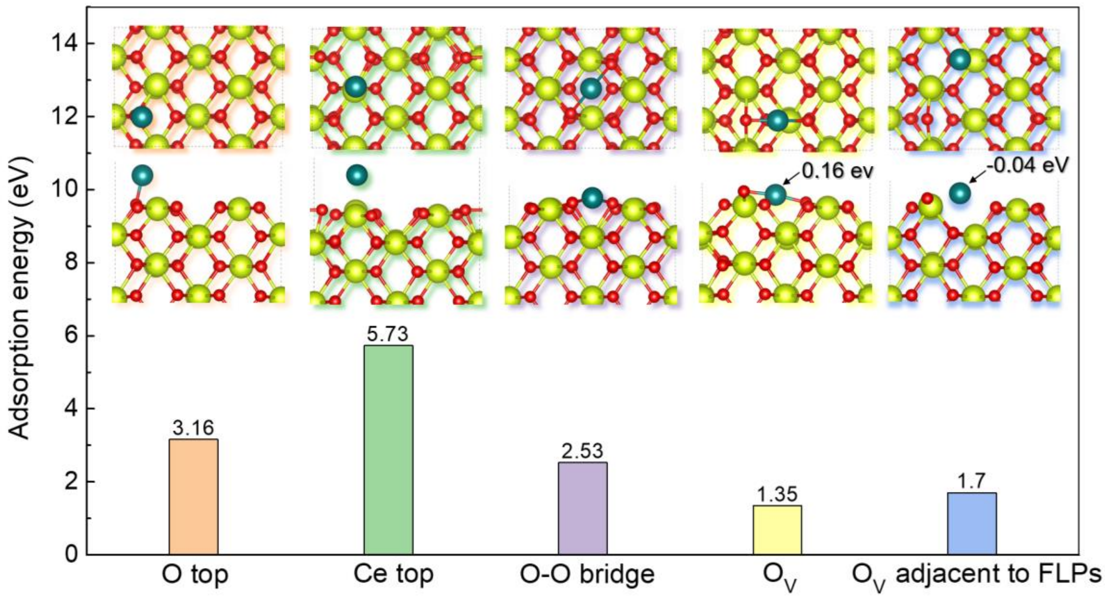

Supplementary Figure 3 | Optimized structure of Pt single-atom on CeO_2(110) surface. Summary of the adsorption energy and spatial configuration of the Pt single-atom at various sites on CeO_2(110) surface.

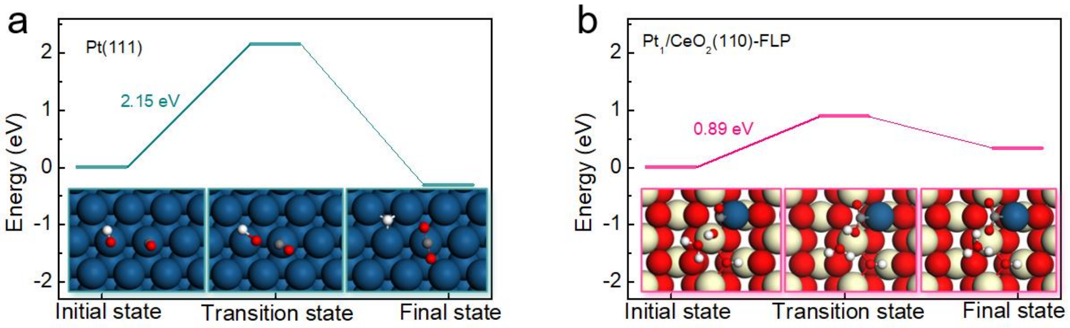

Supplementary Figure 4 | The reforming of *CO with surface hydroxyl group. (**a**) Pt(111) surface and (**b**) Pt_1-FLP dual-active site.

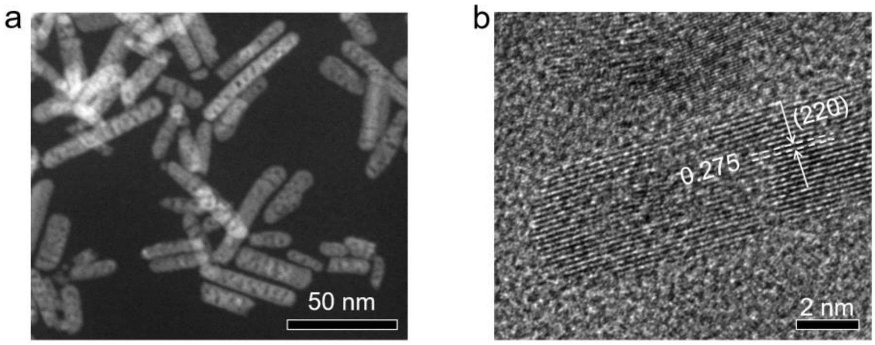

Supplementary Figure 5 | Morphology characterization of PN-CeO2. (**a**) Dark field TEM and (**b**) HRTEM images of the PN-CeO2 catalysts.

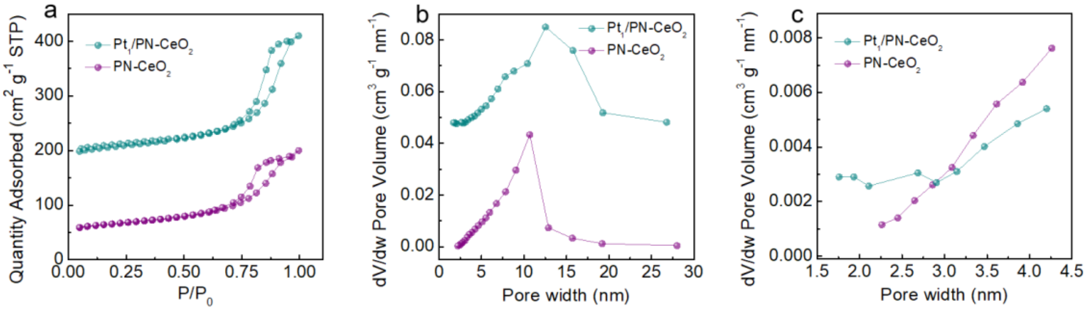

Supplementary Figure 6 | Structural characterization of PN-CeO2 catalysts. (**a**) Nitrogen adsorption/desorption isotherm plot of PN-CeO2. (**b** and **c**) Pore size distribution of PN-CeO2 obtained from BET testing.

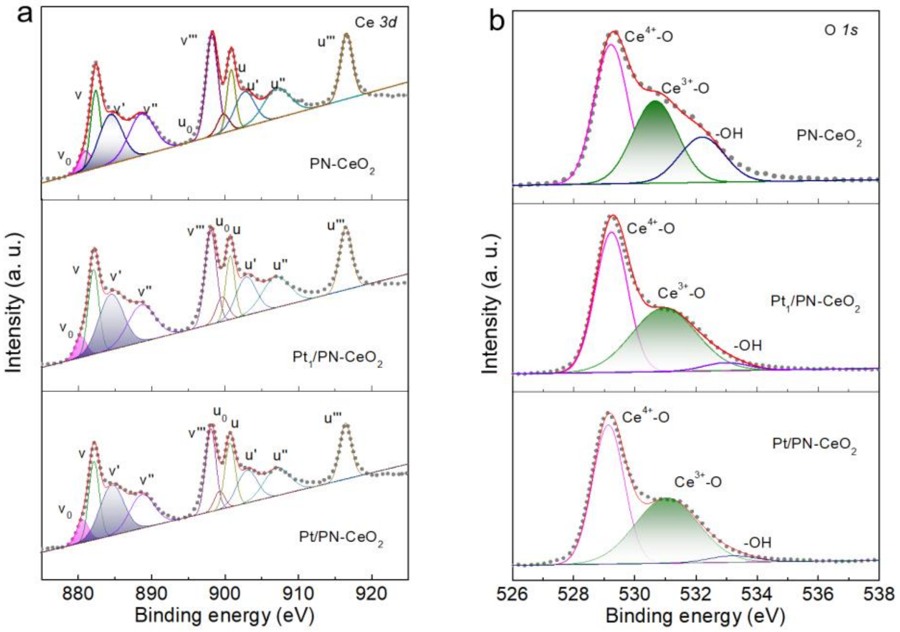

Supplementary Figure 7 | Surface properties of PN-CeO_2 supports. XPS analysis of (**a**) Ce *3d* and (**b**) O *1s* peaks for the PN-CeO_2 catalysts.

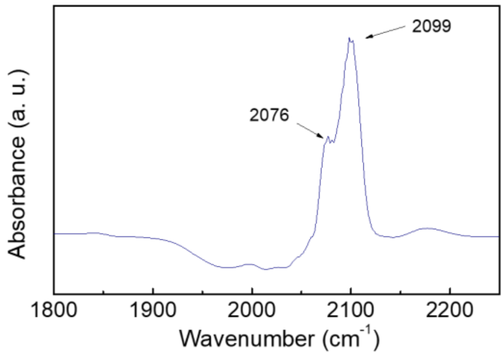

Supplementary Figure 8 | Surface structure of the Pt_1/PN-CeO_2 catalysts. The DRIFTS analysis of the CO adsorption on the surface of Pt_1/PN-CeO_2.

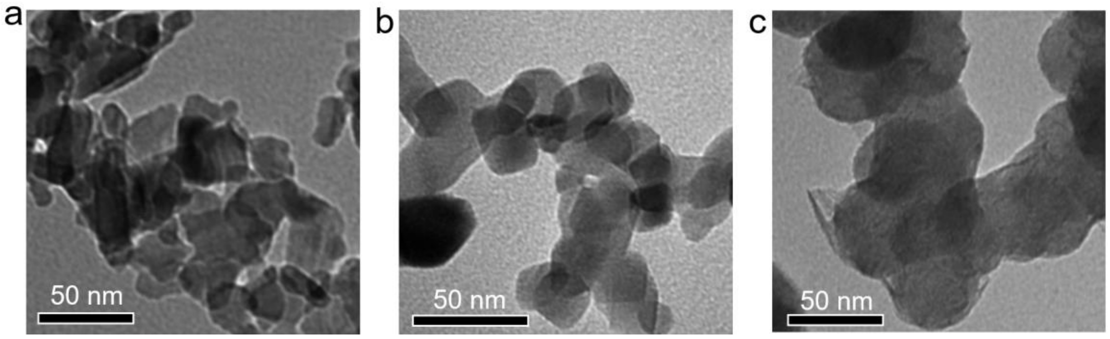

Supplementary Figure 9 | Morphology characterization of various Pt catalysts. TEM images of (**a**) Pt/Al_2O_3, (**b**) Pt/TiO_2 and (**c**) Pt/C catalysts.

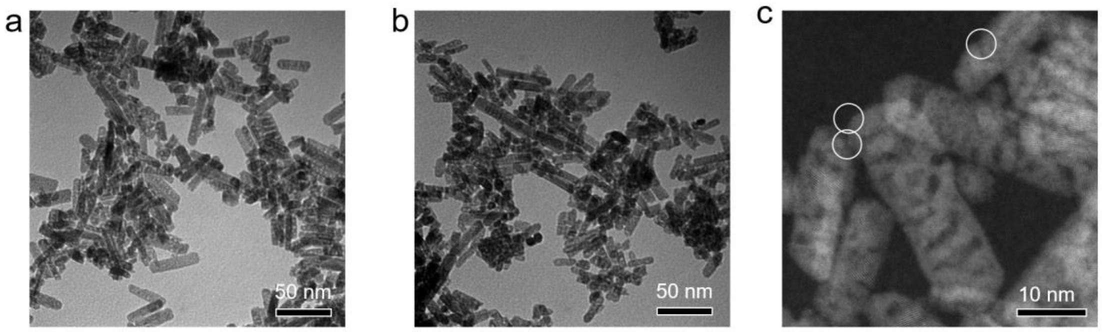

Supplementary Figure 10 | Morphology characterization of used Pt_1/PN-CeO_2. TEM images of the used Pt_1/*PN*-CeO_2 catalysts at (**a**) 165 °C and (**b**) 120 °C, respectively. (**c**) HAADF-STEM image of used Pt_1/PN-CeO_2 at 165 °C.

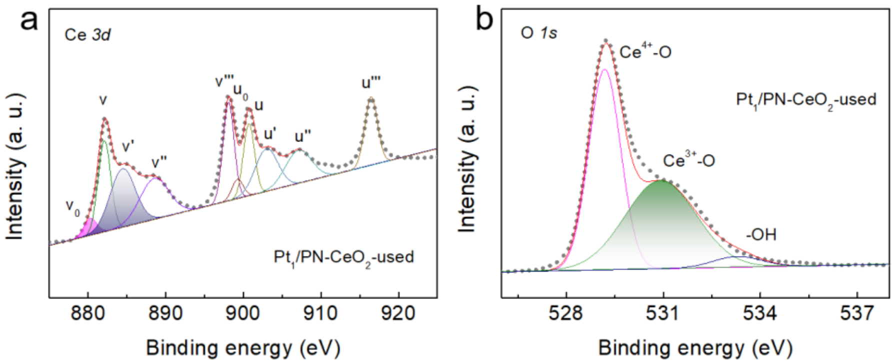

Supplementary Figure 11 | Surface properties of the used Pt_1/PN-CeO_2 catalysts at 165 °C. XPS analysis of (**a**) Ce *3d* and (**b**) O *1s* peaks.

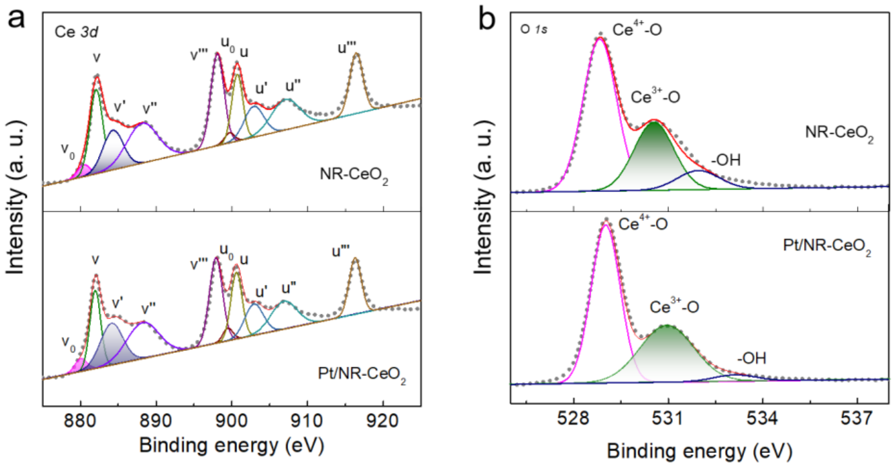

Supplementary Figure 12 | Surface properties of the NR-CeO_2 supports and Pt/NR-CeO_2 catalysts. XPS analysis of (**a**) Ce *3d* and (**b**) O *1s* peaks.

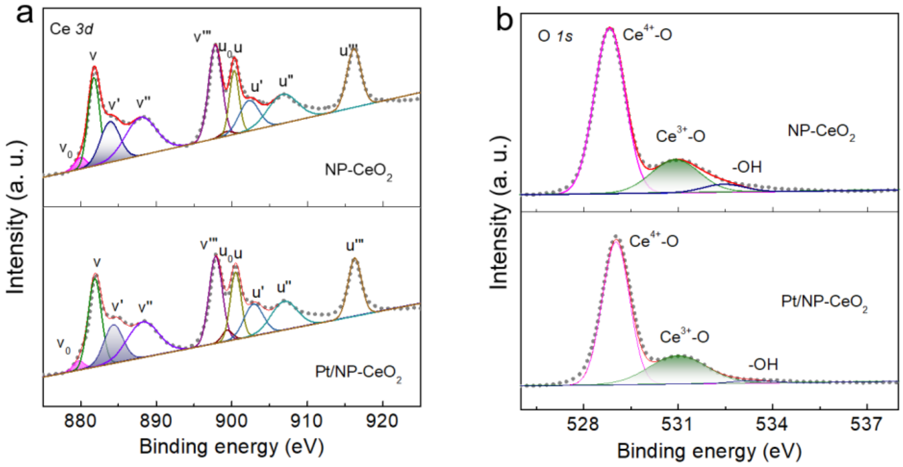

Supplementary Figure 13 | Surface properties of the NP-CeO_2 supports and Pt/NP-CeO_2 catalysts. XPS analysis of (**a**) Ce *3d* and (**b**) O *1s* peaks.

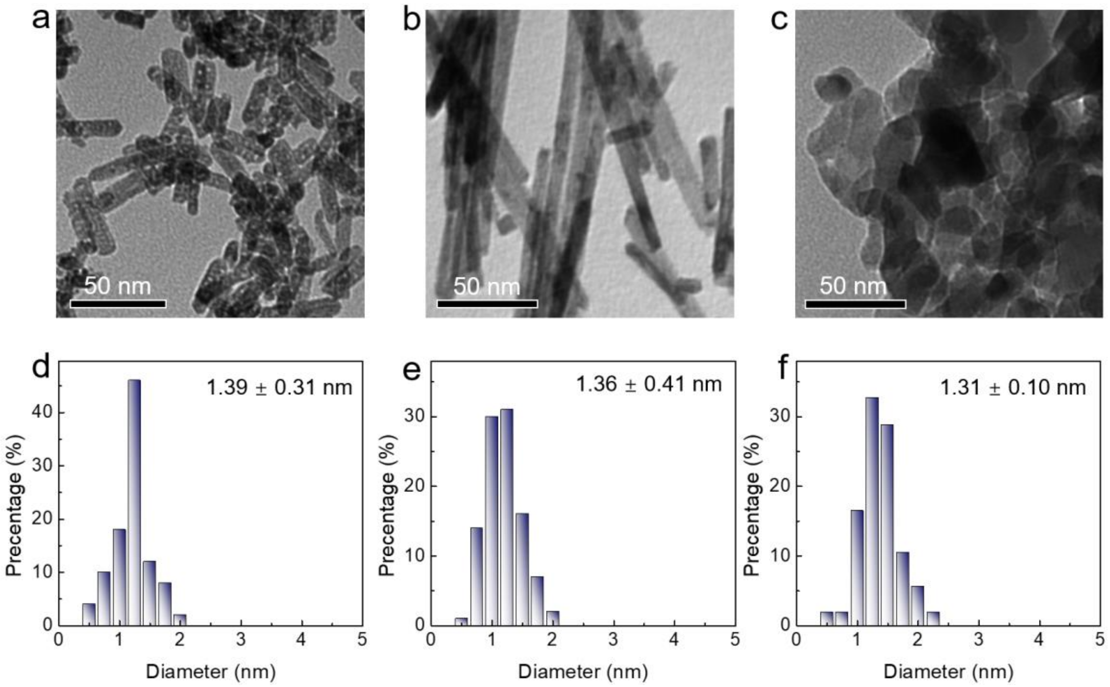

Supplementary Figure 14 | Morphology and size characterization. TEM images of (**a**) Pt/PN-CeO_2, (**b**) Pt/NR-CeO_2 and (**c**) Pt/NP-CeO_2. The size distribution of Pt on the (**d**) Pt/PN-CeO_2, (**e**) Pt/NR-CeO_2 and (**f**) Pt/NP-CeO_2 catalysts.

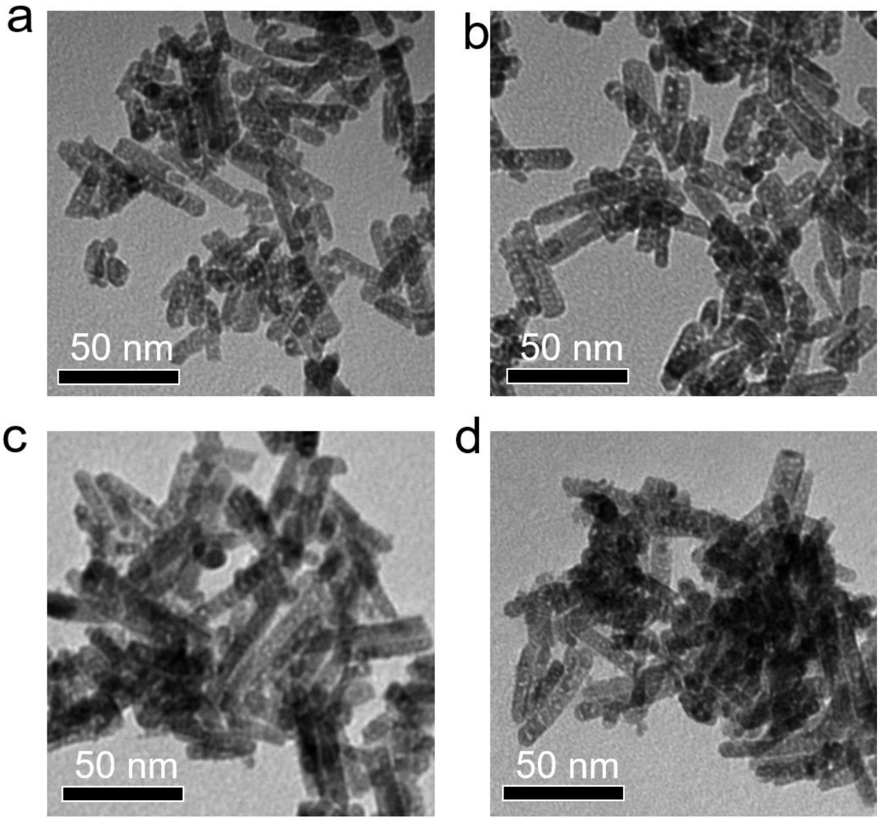

Supplementary Figure 15 | Morphology characterization. TEM images of the Pt/PN-CeO_2 catalysts with Pt loading of (**a**) 0.32 *wt.*%, (**b**) 0.51 *wt.*%, (**c**) 2.1 *wt.*% and (**d**) 5.1 *wt.*%.

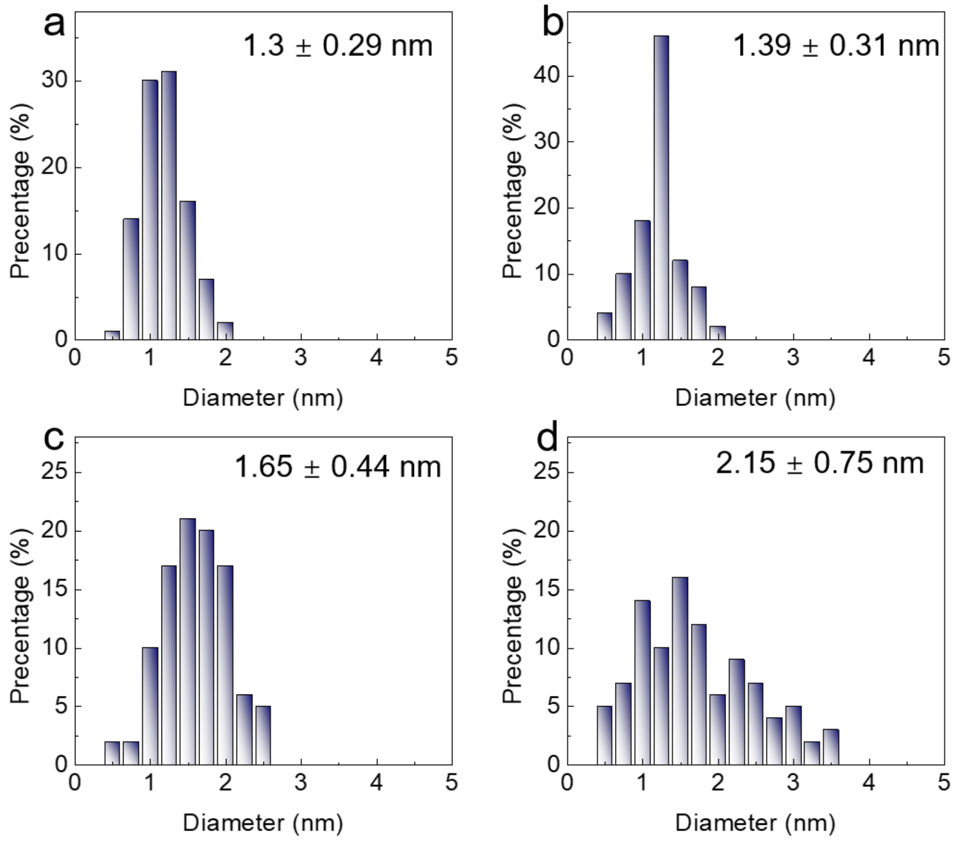

Supplementary Figure 16 | Size characterization. Size distributions of the Pt/PN-CeO_2 catalysts with Pt loading of (**a**) 0.32 *wt.*%, (**b**) 0.51 *wt.*%, (**c**) 2.1 *wt.*% and (**d**) 5.1 *wt.*%.

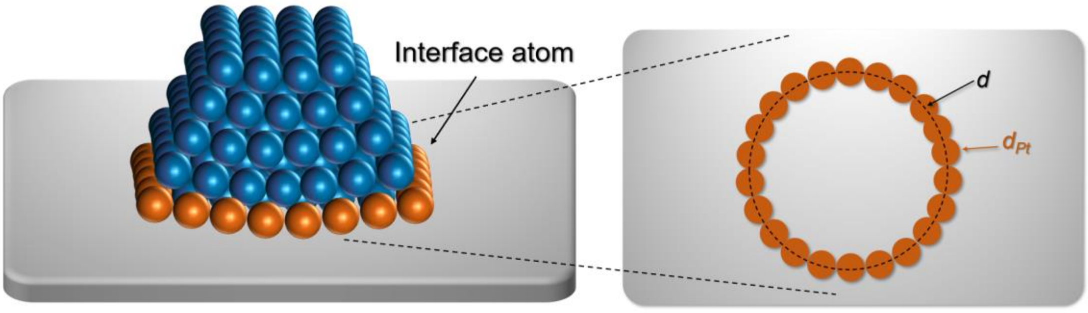

Supplementary Figure 17 | Schematic diagram. Schematic diagram of the supported metal nanoparticles.

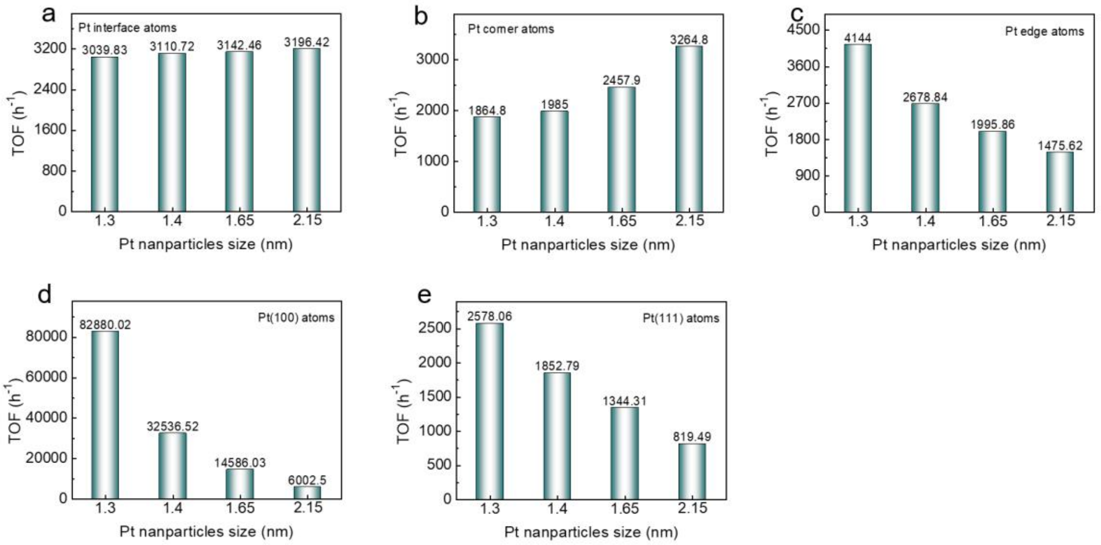

Supplementary Figure 18 | TOF values. The calculated TOF values based on (**a**) interfacial, (**b**) corner, (**c**) edge, (**d**) (100) and (**e**) Pt(111) Pt atoms.

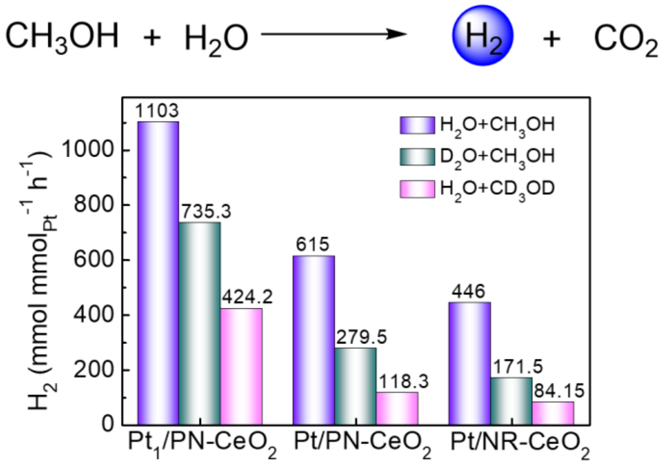

Supplementary Figure 19 | Kinetic isotope effect for H_2 generation from methanol and H_2O. Summary of the H_2 generation rates from CH_3OH/CD_3OD and H_2O/D_2O catalyzed by $Pt_1/PN\text{-}CeO_2$, $Pt/PN\text{-}CeO_2$ and $Pt/NR\text{-}CeO_2$. **Reaction conditions:** catalysts (50 mg), CH_3OH/CD_3OD (40 mL), H_2O/D_2O (18 mL), $n(CH_3OH):n(H_2O)=1:1$, 165 °C and N_2 (0.4 MPa).

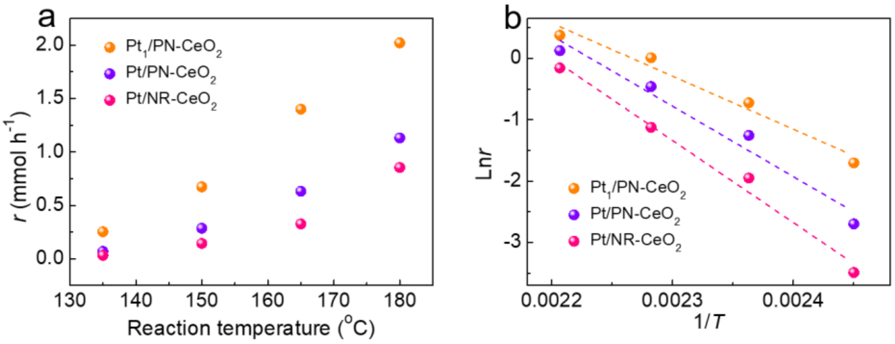

Supplementary Figure 20 | H_2 generation at various reaction temperature. (**a**) H_2 generation rate at various reaction temperature. **Reaction conditions:** catalysts (50 mg), CH_3OH (40 mL), H_2O (18 mL), n(CH_3OH):n(H_2O)=1:1, and N_2 (0.4 MPa). (**b**) Ln*r*, derived from H_2 generation rate versus reaction time, as a function of 1/*T* over various Pt/CeO_2.

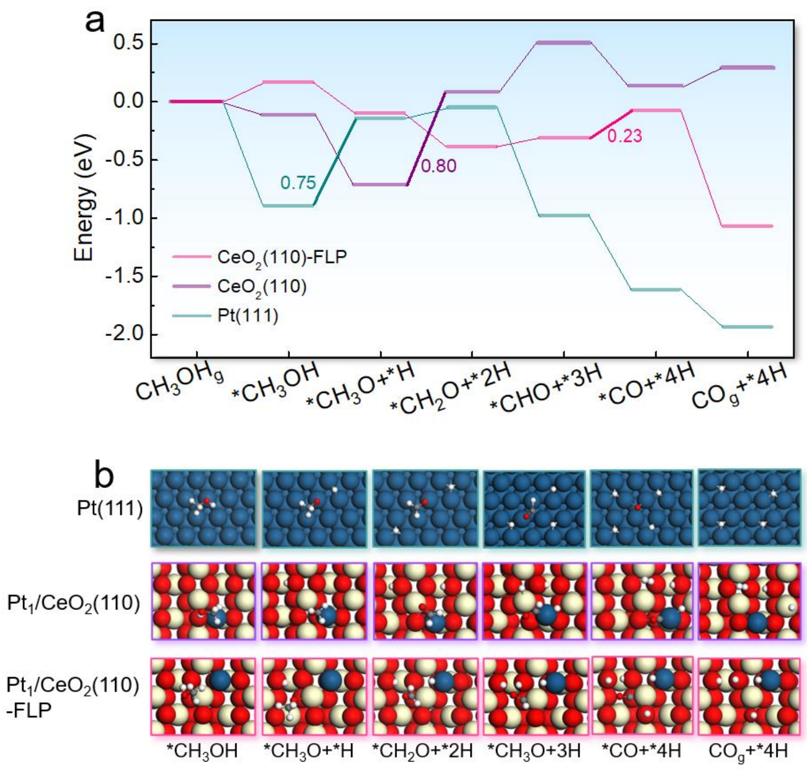

Supplementary Figure 21 | Methanol decomposition on Pt. (**a**) Energy barrier of methanol decomposition on Pt(111), $Pt_1/CeO_2(111)$ and $Pt_1/CeO_2(111)$-FLP. (**b**) The adsorption configuration of various intermediates on Pt(111), $Pt_1/CeO_2(110)$ and $Pt_1/CeO_2(110)$-FLP during the methanol decomposition.

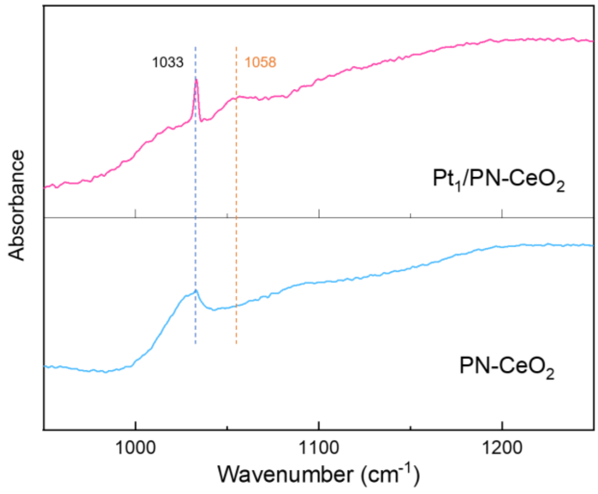

Supplementary Figure 22 | Characterization of surface structure. The FTIR spectrograms of $PN-CeO_2$ and $Pt_1/PN-CeO_2$ after adsorption of methanol.

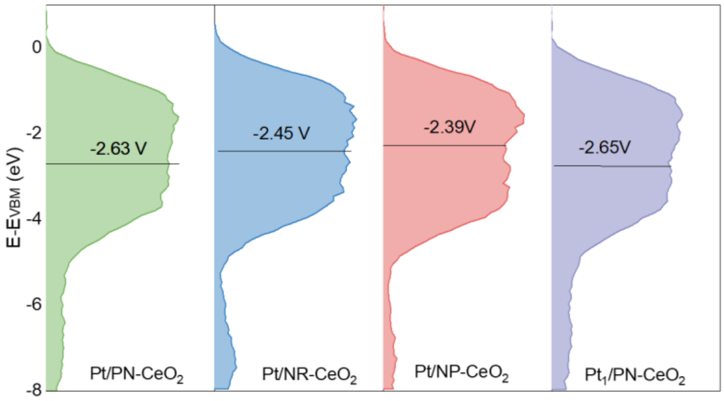

Supplementary Figure 23 | High-resolution valence-band Pt 5d XPS of Pt-*x* relative to the VBM, as an analogue of the density of states. Black lines indicate the positions of d-band centers.

Supplementary References:

1 Perdew, J. P., Burke, K. & Ernzerhof, M. Generalized gradient approximation made simple. *Phys. Rev. Lett.* **77**, 3865 (1996).

2 Lin, J. S., Qteish, A., Payne, M. C. & V. Heine. Optimized and transferable nonlocal separable ab initio pseudopotentials. *Phys. Rev. B* **47**, 4174 (1993).

3 Hu, J., Chen, W., Zhao, X., Su, H. B. & Chen, Z. Anisotropic electronic characteristics, adsorption, and stability of low-index $BiVO_4$ surfaces for photoelectrochemical applications. *ACS Appl. Mater. Interfaces* **10**, 5475-5484 (2018).

4 Wu, X., Selloni, A. & Nayak, S. K. First principles study of CO oxidation on TiO_2 (110): The role of surface oxygen vacancies. *J. Chem. Phys.* **120**, 4512-4516 (2004).

5 Huang, Z. Q., Long, B. & Chang, C. R. A theoretical study on the catalytic role of water in methanol steam reforming on PdZn(111). *Catal. Sci. Technol.* **5**, 2935-2944 (2015).

6 Nielsen, M. *et al.* Low-temperature aqueous-phase methanol dehydrogenation to hydrogen and carbon dioxide. *Nature* **495**, 85-89 (2013).

7 Awasthi, M. K., Rai, R. K., Behrens, S. & Singh, S. K. Low-temperature hydrogen production from methanol over a ruthenium catalyst in water. *Catal. Sci. Technol.* **11**, 136-142 (2021).

8 Andérez-Fernández, M. *et al.* AStable manganese pincer catalyst for the selective dehydrogenation of methanol. *Angew. Chem. Int. Ed.* **56**, 559-562 (2017).

9 Fujita, K., Kawahara, R., Aikawa, T. & Yamaguchi, R. Hydrogen Production from a Methanol-Water Solution Catalyzed by an Anionic Iridium Complex Bearing a Functional Bipyridonate Ligand under Weakly Basic Conditions. *Angew. Chem. Int. Ed.* **54**, 9057-9060 (2015).

10 Monney, A. *et al.* Base-free hydrogen generation from methanol using a bi-catalytic system. *Chem. Commun.* **50**, 707-709 (2014).

11 Hu, P., Diskin-Posner, Y., Ben-David, Y. & Milstein, D. Reusable Homogeneous Catalytic System for Hydrogen Production from Methanol and Water. *ACS Catal.* **4**, 2649-2652 (2014).

12 Rodriguez-Lugo, R. E. *et al.* A homogeneous transition metal complex for clean hydrogen production from methanol-water mixtures. *Nat. Chem.* **5**, 342-347 (2013).

13 Alberico, E. *et al.* Selective hydrogen production from methanol with a defined iron pincer catalyst under mild conditions. *Angew. Chem. Int. Ed.* **52**, 14162-14166 (2013).

14 Yu, J. et al. Effective low-temperature methanol aqueous phase reforming with metal-free carbon dots/C_3N_4 composites. *ACS Appl. Mater. Interfaces* **13**, 24702-24709 (2021).
